# Evaluating generalization in protein–ligand cofolding methods

**DOI:** 10.1038/s41594-026-01797-5

**Published:** 2026-05-08

**Authors:** Peter Škrinjar, Jérôme Eberhardt, Gabriel Studer, Gerardo Tauriello, Torsten Schwede, Janani Durairaj

**Affiliations:** 1 Biozentrum, University of Basel, Basel, Switzerland; 2 SIB, Swiss Institute of Bioinformatics, Basel, Switzerland

**Keywords:** Computational biology and bioinformatics, Structural biology

## Abstract

Deep learning has driven major breakthroughs in protein structure prediction; however, one of the next critical steps forward is accurately predicting how proteins interact with small-molecule ligands, to enable real-world applications such as drug discovery. Recent cofolding methods aim to address this challenge, but evaluating their performance has been inconclusive because of the lack of relevant benchmarking datasets. Here we present a comprehensive evaluation of four leading all-atom cofolding methods using our newly introduced benchmark dataset, Runs N’ Poses. Runs N’ Poses comprises 2,600 high-resolution protein–ligand systems released after the training cutoff used by these methods. We demonstrate that current cofolding approaches largely memorize ligand poses from their training data, hindering their use for de novo drug design. With this assessment and benchmark dataset, we aim to accelerate progress in the field by allowing for a more realistic assessment of the current state-of-the-art deep learning methods for predicting protein–ligand interactions.

## Abstract (web summary)

This work introduces the Runs N’ Poses dataset for benchmarking deep learning methods on the protein–ligand complex prediction task. It shows that current methods rely on memorization, challenging their effectiveness for real-world applications such as drug discovery.

## Main

Being able to accurately predict and characterize protein–ligand interactions (PLI) is a cornerstone of modern drug discovery, enabling the identification of small molecules that can modulate protein function with high specificity. The process of discovering and optimizing such modulators into viable lead compounds is a critical aspect of early-stage pharmaceutical development and lead optimization. Structure-based drug design, a component of computer-aided drug design, relies on three-dimensional structures of the target protein and the protein–ligand interface to inform medicinal chemistry decisions^1–3^. Therefore, to achieve accurate predictions of how ligands bind and their binding affinity, a deep understanding of the fundamental principles governing PLIs is essential^4^. A combination of different computational techniques has become indispensable in both industrial and academic drug-discovery efforts, helping to reduce costs and accelerate development timelines^5^. However, despite substantial advances in computational methods, accurately modeling protein–ligand binding at atomic resolution remains an important challenge^6,7^.

Over the years, there have been numerous community initiatives to assess the state of the field in predicting the structure of ligand poses binding to proteins. Recently, CASP15 had a pilot experiment in the evaluation of protein–ligand complex prediction^8^, inspired by previous efforts such as Teach Discover Treat^9^, Continuous Evaluation of Ligand Prediction Performance^10^, Drug Discovery Data Resource^11–14^ and Community Structure–Activity Resource^15,16^, with the added complexity that the prediction task consisted of predicting both the structure of the receptor protein and the position and conformation of one or more ligands. Conclusions from CASP15^17^ and parallel benchmarking efforts^18^ were threefold. First, the joint protein–ligand prediction task requires accuracy metrics that take both protein and ligand positioning into account, such as binding-site superposed root mean square deviation (r.m.s.d.) and local difference distance test (LDDT) of protein–ligand interactions (LDDT-PLI)^17^. Second, experimental quality is a more acute issue for PLI prediction compared to protein structure prediction, owing to the atom-level reliability required and the fact that many Protein Data Bank (PDB)^19^ entries fail to meet even relaxed X-ray quality standards^18,20^ to enable their use as reliable ground truth. Third, many complexes with ligands released in the PDB are very similar to previously released complexes or not relevant for drug-discovery applications, or both. Although CASP16 addressed part of the latter issue by soliciting unreleased PLI complexes from pharmaceutical companies, the scarcity of targets and the availability of very similar templates in the PDB make it difficult to draw conclusions specifically about deep learning methods that qualify for this task, most of which also did not participate.

Deep learning methods such as AlphaFold2^21^ have revolutionized protein structure prediction, inspiring the development of all-atom cofolding methods like RoseTTAFold All-Atom^22^, AlphaFold3^23^, Chai-1^24^, Boltz-1^25^, Boltz-2^26^, Protenix^27^ and NeuralPLexer3^28^. Although these methods show promise, evaluating their performance is difficult because of the lack of established benchmarking techniques for joint protein–ligand binding in the deep learning domain. Existing benchmarks often fail to account for similarities between the training data and test sets, leading to inflated performance estimates^18,20,29,30^. This is particularly problematic for PLI systems, where the scarcity and bias in experimental structures can exacerbate both the risk of memorization and our inability to detect it.

To address this, we present a comprehensive benchmark of four leading all-atom cofolding deep learning methods with highly similar architectures and training paradigms (AlphaFold3^23^, Chai-1^24^, Protenix^27^ and Boltz-1^25^) on 2,600 high-resolution PLI systems released after their training cutoff (30 September 2021). We explore the impact of training data similarity on prediction accuracy, revealing a critical limitation: current cofolding methods struggle to generalize beyond ligand poses seen in their training data. We also compared the top-ranked and best-scored models, observing a slight improvement in performance, yet the overall trend of limited generalization remained unchanged.

This overfitting issue, independent of model confidence scores, highlights the need for improved architectures and data augmentation strategies. Notably, we observe better generalization for cofactor, amino acid and nucleotide analogs, suggesting the importance of abundant training data. We provide a benchmark dataset (Runs N’ Poses) with varying degrees of training set similarity, along with resources to analyze the relationship between data leakage and performance. With this work, we aim to facilitate progress and enable identification of genuine breakthroughs in the critical field of protein–ligand complex prediction.

## Results

### Limited generalization of cofolding methods across protein–ligand complex space

**Fig. 1:**
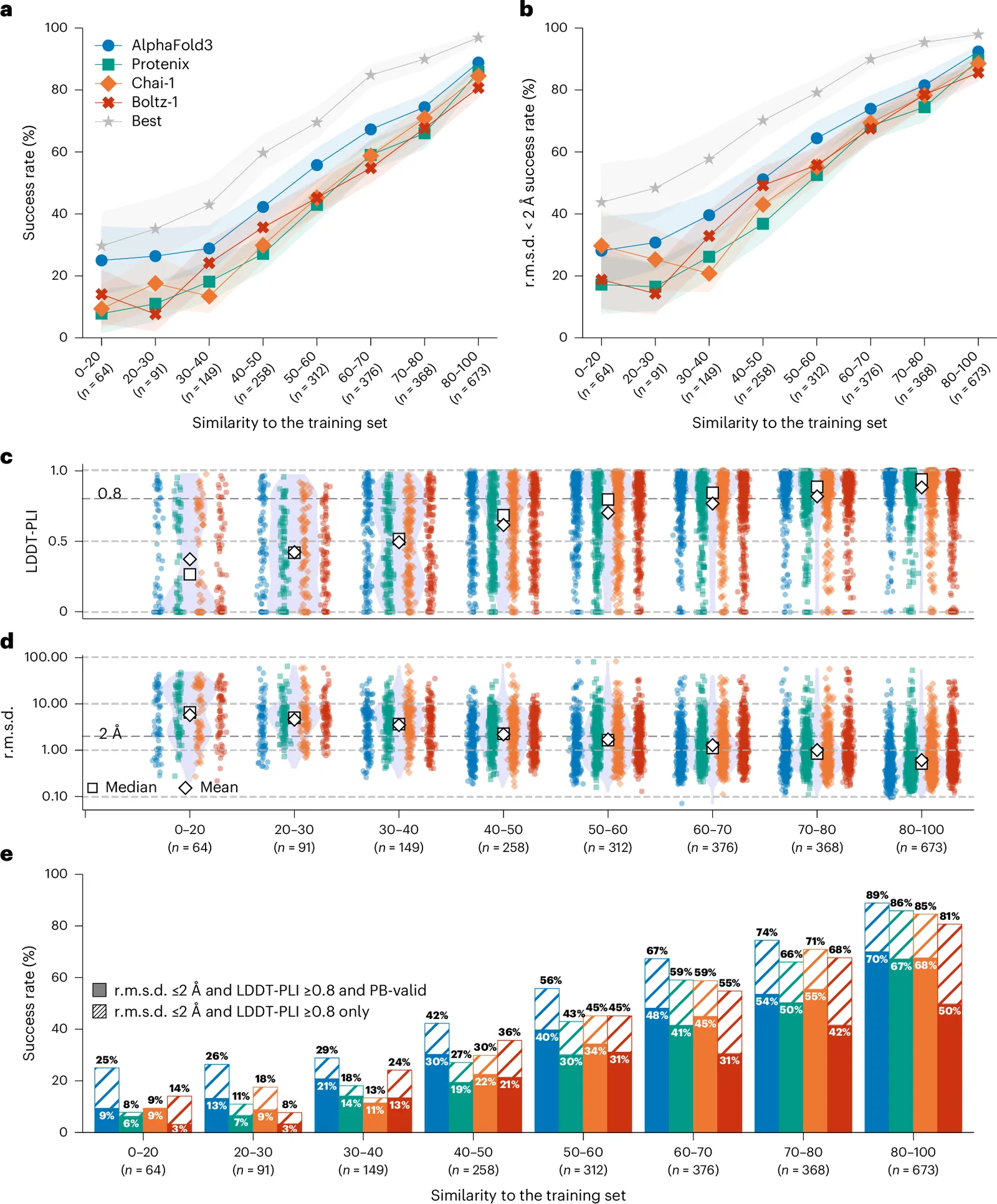
Prediction accuracy versus training set similarity. **a**, Success rate, defined as the percentage of system ligands with <2 Å r.m.s.d. and >0.8 LDDT-PLI, for all common subset system ligands in each similarity bin across the four methods and when selecting the best-scored model from the four top-ranked. Shaded regions correspond to the 95% confidence interval, calculated from 1,000 bootstrap samples for each bin and method. **b**, As **a** but with the success rate defined only by <2 Å r.m.s.d. **c**,**d**, The distribution of LDDT-PLI (**c**) and r.m.s.d. (**d**) values for all methods displayed as a violin plot, with each individual method being shown as colored scatter points. **e**, As **a** with added physical validity checks. Striped bars show the share of predictions of each method that have <2 Å r.m.s.d. and >0.8 LDDT-PLI and solid bars show the subset that in addition have valid geometries and energies; that is, pass all PoseBusters tests and are therefore ‘PB-valid’.

We aimed to explore the dependence of prediction accuracy on the similarity of the target complex to the training set for the four deep learning cofolding methods, based on our benchmark dataset of 2,600 systems (covering 3,047 nonion nonartifact ligands). Our analysis shows a clear correlation between the accuracy of the top-ranked predicted model for each method and the similarity of the reference system to the training set (Fig. 1a).

Here we define the similarity to the training set as the product of binding-pocket coverage^30^ and Combined Overlap Score (SuCOS) of the ligand pose that measures the extent to which ligand poses overlap in both volume and placement of chemical features^31–33^. Thus, a training system is only considered similar if it has a similar ligand pose in a similar protein pocket to the test system (further details on the protein and ligand similarity metrics are given in the section ‘Ligand and protein similarity metrics for detecting data leakage’).

Moreover, in classical docking benchmark studies, success is typically defined solely by ligand r.m.s.d. (<2 Å), because the protein structure is generally treated as rigid and assumed to be correct. However, for cofolding methods, it is essential to assess the accuracy of both the ligand and the protein structure. Therefore, we define the success rate as a combination of LDDT-PLI (>0.8) and ligand pose r.m.s.d. (<2 Å)^34^ (further details on this choice are described in the ‘Accuracy scoring’ section of the Methods). Figure 1b, where success is defined solely on r.m.s.d. (<2 Å), confirms that our success rate criteria do not influence the observed trend of limited generalization. As shown in Fig. 1c,d, we observed that LDDT-PLI and r.m.s.d. distributions for each method consistently correlate with the similarity to the training set.

Finally, Fig. 1e shows the fraction of predictions passing physical validity checks as defined by the PoseBusters (PB) suite^20^. PoseBusters validity appears to be independent of training set similarity, with roughly the same fractions seen for systems with low and high training set similarity across all four methods (percentage of successful PB-valid of all successful systems is 67.2 ± 7.4 in the 0–50 similarity bin and 72.2 ± 6.5 in the 50–100 bin).

### Model and data choices lead to marginal performance improvement

To directly compare methods to each other, Fig. 1 focused on the common subset of systems that were predicted by all four methods using their default settings, ignoring cases in which certain methods fail to produce an output because of varying steps in their data processing and prediction pipelines (see Table 2 and Methods for statistics on the method-specific failures). Although all four methods exhibit comparable correlations of accuracy with their training set similarity, AlphaFold3 demonstrates a slight performance advantage over the others, despite all methods sharing similar architectures and training paradigms. This advantage may stem from differences in training times, dataset sampling strategies, validation sets and other methodological choices between the four methods. However, as indicated by the gray line representing the best-scored of the top-ranked models across all four methods (Fig. 1a,b), AlphaFold3 is not the best-performing method on every system evaluated.

The panels in Supplementary Fig. 1a–d are the same as in Fig. 1a–d, but calculated on all predictions (ignoring method-specific failures), and in addition show the results from: (1) AlphaFold3 run without templates (light blue), (2) Boltz-1x (pink) and (3) RoseTTAFold All-Atom^22^ (purple). AlphaFold3 with and without templates does not show any significant differences; this is expected because ligand information from the template is not utilized by the method. RoseTTAFold All-Atom has a different architecture and was run with only 1 model output instead of 25 and thus is not directly comparable; however, this default setting performed poorly compared to the others.

Interestingly, Boltz-1x does not show differences in success rate compared to Boltz-1 v.0.4, but has a boost in PoseBusters physical validity checks (Supplementary Fig. 1e,f). Supplementary Fig. 1e shows the fractions of systems failing each individual check for all cofolding methods. The most problematic checks are the minimum_distance_to_protein check, which passes if the distance between protein–ligand atom pairs is >0.75 times the sum of the pairs van der Waals radii, with 23.4% of predicted models failing this check across the four methods; and the tetrahedral chirality check, which passes if the specified tetrahedral chirality in the input molecule is the same as in the true molecule, with 16% of predicted models failing this check across the four methods. Failing the former check suggests physically implausible positioning of atoms that would lead to steric clashes between the protein and the ligand, while failing the second results in a predicted molecule with a different stereochemistry than the input chemical description. Both would result in models effectively unusable for downstream analysis of interactions and binding. However, as shown in the Boltz-1x column of Supplementary Fig. 1e, most failing cases are solved by Boltz-1x. Boltz-1x adds inference time diffusion steering to the base Boltz-1 model^25^, with physics-inspired potentials specifically aimed to address issues with physical reliability. Such a steering approach can in principle be applied to any cofolding model relying on diffusion, and thus physical plausibility of poses can be greatly improved. Nevertheless, as shown in Supplementary Fig. 1f, this does not affect the memorization trend.

**Fig. 2:**
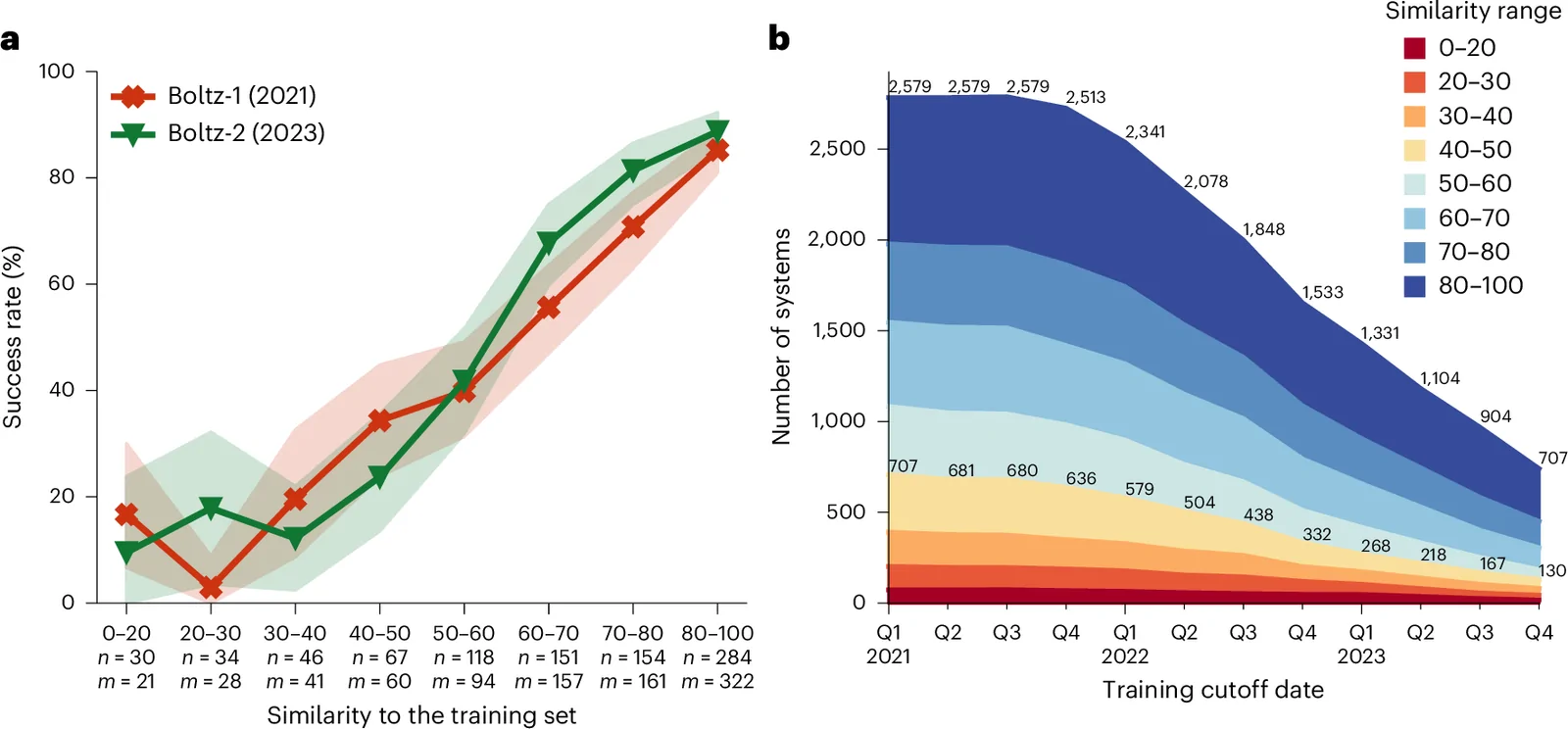
Benchmarking with different training cutoffs. **a**, Success rate, defined as the percentage of system ligands with <2 Å r.m.s.d. and >0.8 LDDT-PLI, for systems released after the Boltz-2 training cutoff when predicted by Boltz-1 (red) and Boltz-2 (green) for each similarity bin. The similarity bins are calculated using the 30 September 2021 training cutoff for Boltz-1 (counts labeled as *n*), and the 1 June 2023 training cutoff for Boltz-2 (counts labeled as *m*). Shaded regions correspond to the 95% confidence interval, calculated from 1,000 bootstrap samples for each bin and method. **b**, Training set similarity distributions for different training cutoffs. Counts of systems with <50 similarity and the total number of systems released after the cutoff are labeled. Q, quarter.

Since the creation of Runs N’ Poses, Boltz-2^26^ has been released, which was trained with a cutoff of 1 June 2023, thus using a full two years more PDB data than the other methods. Although this makes the new method not directly comparable with the others presented here, it can still be assessed using the similarity to the training set. We compared Boltz-2 performance on Runs N’ Poses systems released after the 2023 training cutoff, as a function of the similarity to the PDB up to this point, with the Boltz-1 performance with similarities calculated according to the Boltz-1 training cutoff. As seen in Fig. 2a, the additional data used for training (more than 25,000 PDB entries) increases the number of cases in the higher similarity bins but does not seem to help with generalization to unseen data.

Figure 2b demonstrates the number of systems available in Runs N’ Poses for various training cutoffs as well as their distribution across the different similarity bins. Although there is still a reasonable number of cases for benchmarking even post 2023, there is a significant decrease in cases in the more difficult bins as more diversity is added to the PDB, lowering the capability to perform granular and confident analysis of memorization and generalizability.

### Impact of ligand and complex prevalence

**Fig. 3:**
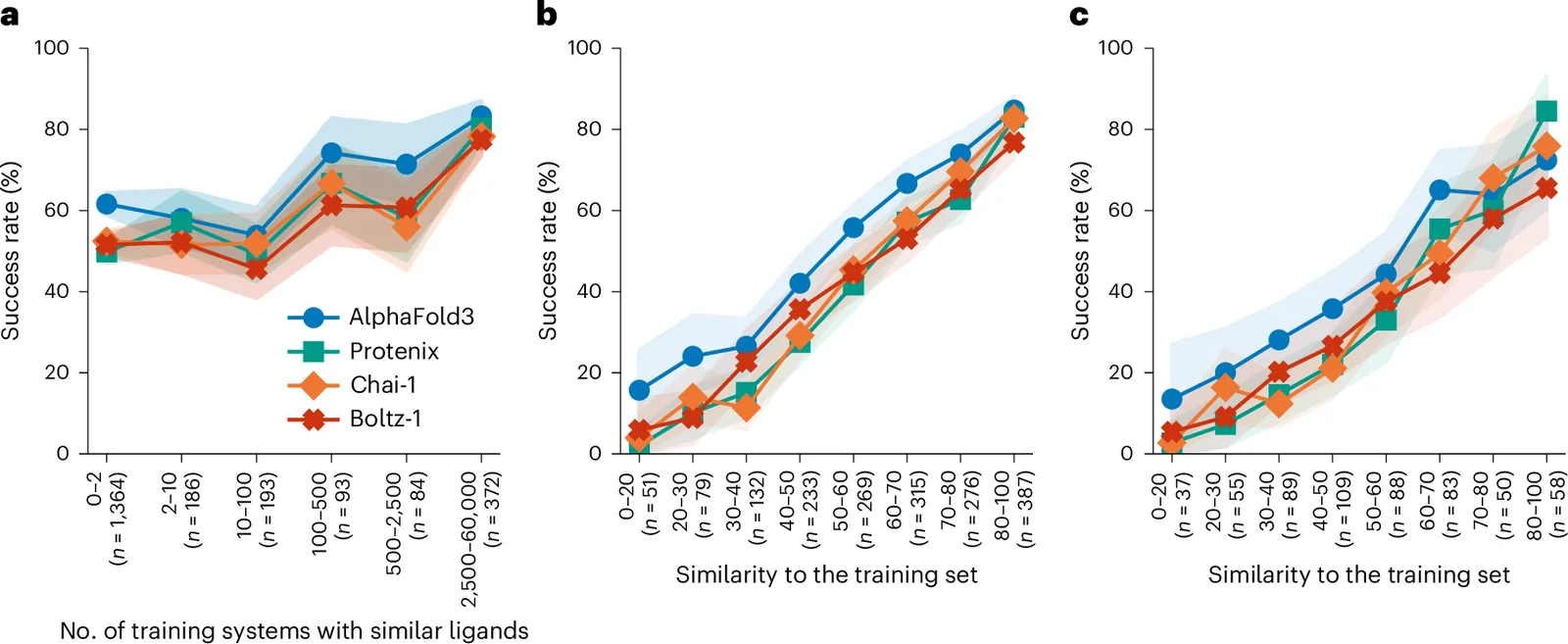
Prediction accuracy for prevalent versus distinct ligands. **a**–**c**, Success rate, defined as the percentage of system ligands with <2 Å r.m.s.d. and >0.8 LDDT-PLI across the four methods, for system ligands with differing numbers of analogous ligand training systems (**a**), only system ligands having <100 analogous ligand training systems across each similarity bin (**b**) and only cluster representatives from **b** across each similarity bin (**c**). Shaded regions correspond to the 95% confidence interval, calculated from 1,000 bootstrap samples for each bin and method.

Further stratification reveals slightly improved performance on ligands with substantial amounts of training data (Fig. 3a). This includes cofactors and their analogs, because these are found across a wide variety of protein families and thus also across protein–ligand structures and pockets in the PDB. It also includes analogs of amino acids and nucleotides, likely a benefit of training on protein–protein and protein–nucleotide complexes. To set these apart from more drug-like molecules, we classify them based on their frequency of occurrence in the PDB, independent of the protein and pocket. Specifically, for each ligand in the test set we counted the number of times any analogous ligand appears in any system in the training data. We define analogous ligands as those with >0.9 ligand RDKit topological fingerprint Tanimoto similarity^35^ to the query ligand. Supplementary Fig. 2 depicts molecules with more than 100 analogous occurrences, hereafter referred to as prevalent. Removing these ligands from consideration, keeping only distinct ligands, shows a more linear trend of similarity to success rate (Fig. 3b), demonstrating that the successes in the lower similarity bins are primarily due to such cases. This analysis indicates that data scarcity is one of the drivers of memorization, because drug-like molecules typically do not have a lot of experimental structures resolved in different protein pockets.

We verified that the observed trend is not driven by an over-representation of certain protein families, which could bias the results. Figure 3c displays the same trend correlating success versus training set similarity only for the cluster representatives of distinct ligand systems, based on graph community clustering at SuCOS–pocket similarity >50. This leads to a total of 789 clusters in the common subset of 1,017 total clusters. Supplementary Table 1 details the sizes, cluster representative details and similarity bin coverage of the ten biggest clusters. The first cluster consists of 171 crystal structures of SARS-CoV2 main protease in complex with different small molecules from independent efforts such as the SARS-CoV2 moonshot series^36^ and others^37^, while the second consists of 136 protein kinases complexes with various inhibitors. Thus SuCOS–pocket clustering puts together protein–ligand complexes in which similar molecules bind similar proteins in the same pocket and with similar three-dimensional poses. For example, the HCov-NL63 main protease complexes in cluster 4 have different binding modes from those in cluster 1. Although Fig. 3c shows that the memorization trend is independent of redundancy, such congeneric series—ligands sharing the same core structure and differing only in some substituent groups—are of high relevance to drug-discovery applications and accuracy across these series is an important target to aim for.

We also show that the methods are not biased in performance toward smaller or larger ligands (Supplementary Fig. 3a,b). Although we observed a slight correlation between success rate and binding-pocket size (Supplementary Fig. 3c), the lack of generalization trend is still seen across different binding-pocket sizes (Supplementary Fig. 3d–h). Moreover, looking at different subsets of the dataset, such as single-ligand, multi-ligand and multi-protein systems only, shows again the same pattern, demonstrating the effectiveness of our dataset for benchmarking these sub-tasks (Supplementary Fig. 3i–k).

### Ligand and protein similarity metrics for detecting data leakage

**Fig. 4:**
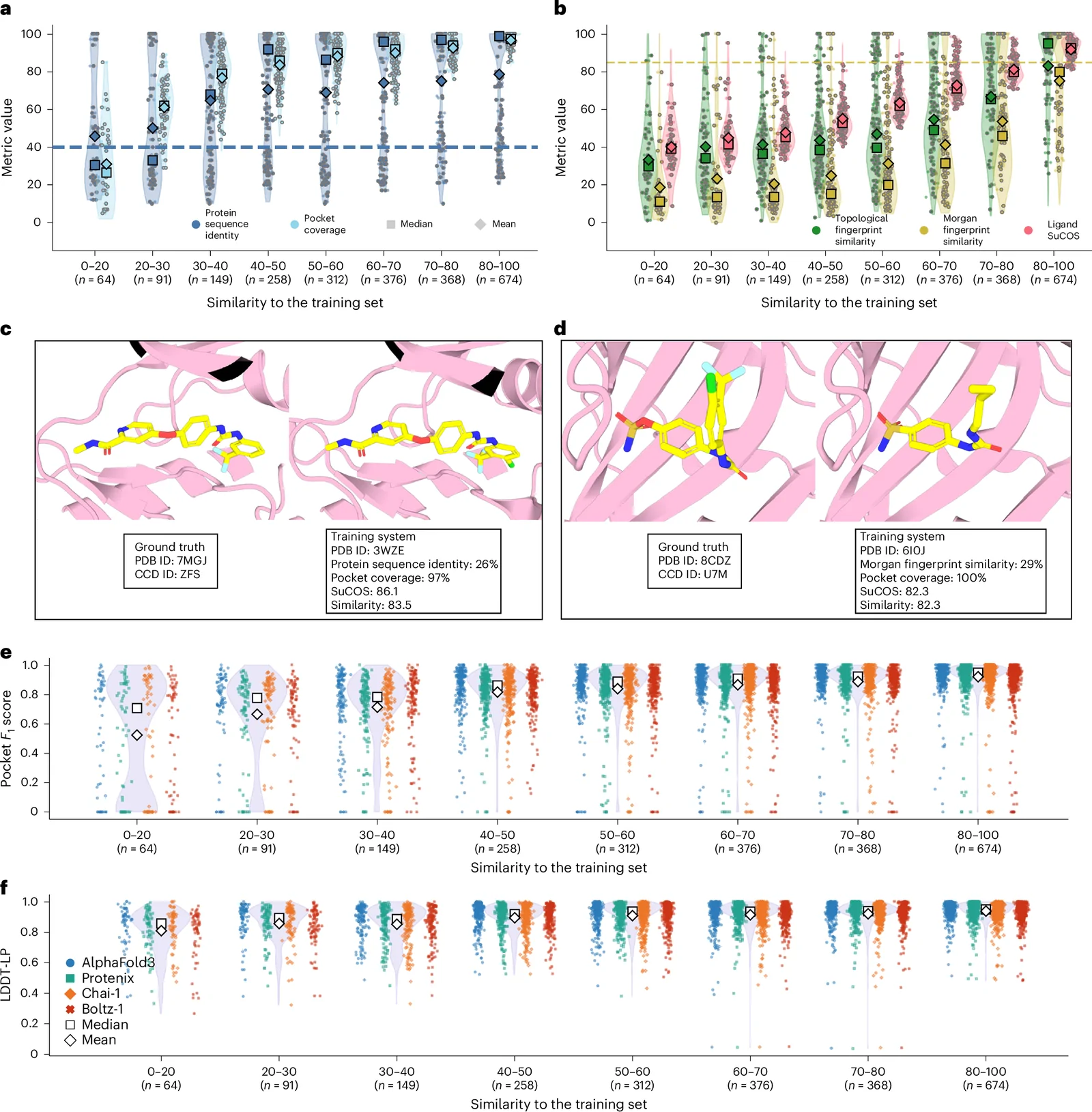
Comparison with other similarity and accuracy metrics. **a**, Distribution of protein sequence identity and pocket coverage (both shown as a percentage) for the closest training system found by SuCOS–pocket similarity. The dashed line indicates the protein sequence identity threshold used in validation of AlphaFold3. **b**, Distribution of ligand topological fingerprint Tanimoto similarity, ligand Morgan fingerprint Tanimoto similarity (radius = 2, *n* bits = 2,048) and ligand SuCOS similarity for the closest training system found by SuCOS–pocket similarity. The dashed line marks the ligand Morgan fingerprint Tanimoto similarity threshold used in validation of AlphaFold3. **c**, Example complex with low sequence identity and high SuCOS–pocket similarity to the closest training system. **d**, Example complex with low Morgan fingerprint similarity and high SuCOS–pocket similarity to the closest training system. **e**, *F*_1_-score distribution for recovering binding-pocket residues across similarity bins for all methods (gray violin) and each method separately (colored scatter). **f**, Distribution of LDDT-LP values across similarity bins for all methods (gray violin) and each method separately (colored scatter). Percentage of systems with LDDT-LP >0.8. For all systems: AlphaFold3, 92%; Protenix, 91%; Chai-1, 90%; Boltz-1, 91%. For cluster representatives (gray outlines): AlphaFold3, 87%; Protenix, 83%; Chai-1, 82%; Boltz-1, 83%. CCD, Chemical Component Dictionary.

To explore how much the memorization trend is influenced by similarity at protein, pocket and ligand levels, Fig. 4a,b shows how protein sequence identity, pocket coverage, ligand SuCOS and ligand fingerprint similarity (using the RDKit topological fingerprint and Morgan fingerprint with radius 1 and 2,048 bits) evolve across similarity bins. This reflects the relationship between each system and its closest training system as determined by SuCOS–pocket similarity. Here the relationship between the SuCOS score and pocket coverage becomes clearer, because the lowest bin (SuCOS–pocket similarity between 0 and 20) includes examples with high SuCOS but low pocket coverage, and vice versa. By contrast, protein sequence identity and ligand fingerprint similarity vary more broadly, but also tend to increase with increasing SuCOS–pocket similarity. We conclude that previous similarity metrics and thresholds used for validating cofolding methods’ performance are insufficient to detect data leakage for the PLI prediction task. The commonly used 40% sequence identity threshold misses numerous cases of proteins from the same family sharing the same fold, pocket and binding mode, and the 85% Morgan fingerprint similarity threshold (used for validation of AlphaFold3^23^) misses cases of ligands binding with highly similar poses to highly similar proteins and pockets, as exemplified in Fig. 4c,d.

Interestingly, already from the third bin (SuCOS–pocket similarity between 30 and 40) we see that the protein and pocket of a test system share more than 60% sequence identity and 80% pocket coverage with the closest training system on average. This is further supported in Fig. 4e, which shows the *F*_1_-score distribution for recovering binding-pocket residues across different similarity bins. Although there is a correlation with similarity to the training set, finding the correct pocket does not seem to be the limiting factor for inaccurate complex predictions. Thus, the trend that we observe is not a case of lack of out-of-distribution generalization for lesser-studied proteins or novel pockets, and more likely arises from other factors, such as the difficulty in generalizing to unseen ligand poses in otherwise familiar pockets.

To illustrate binding-pocket conformation prediction, Fig. 4f shows the per-similarity bin distribution of the local difference distance test of the ligand pocket (LDDT-LP); that is, the accuracy of the relative positioning of residues in the binding pocket. Although there is some increasing trend across the bins, the majority of predictions have an LDDT-LP value >0.8, indicating that the protein is well-modeled. However, these results do not guarantee that the predicted protein models could be used for downstream docking applications, because previous studies showed that even LDDT-LP values >0.9 do not detect atom-level conformational changes that lead to unsuccessful rigid docking^18^.

**Fig. 5:**
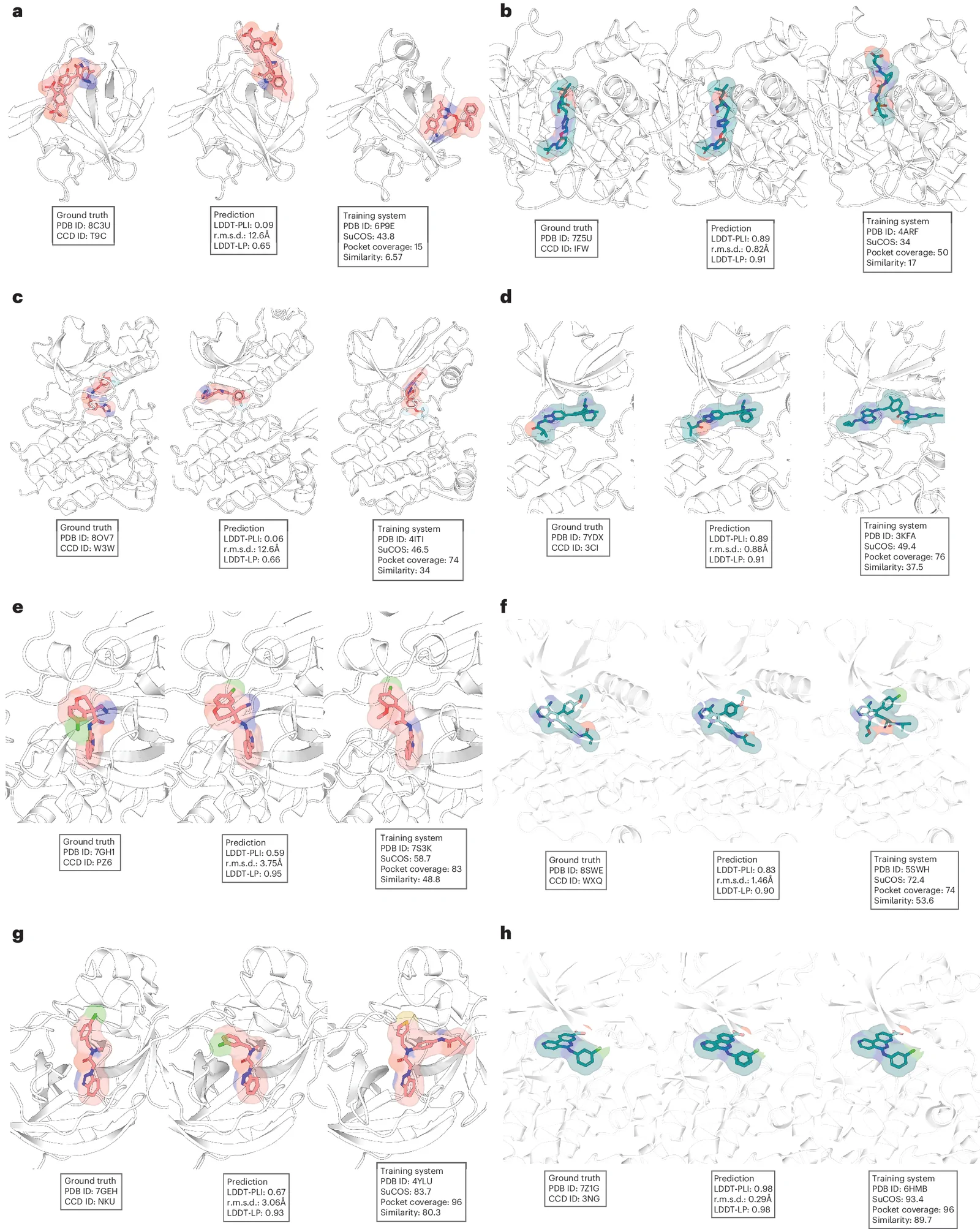
Examples of incorrect and correct predictions across different similarity bins. **a**–**h**, Predicted models from AlphaFold3 (middle) compared to the ground truth experimental structure (left) and the closest training system by SuCOS–pocket similarity (right). The PDB ID and CCD code of the ground truth, the r.m.s.d., LDDT-PLI and LDDT-LP of the prediction, and the PDB ID, SuCOS score, pocket coverage (percentage) and SuCOS–pocket similarity score of the closest training system are labeled. We depict an incorrect prediction (red, **a**,**c**,**e**,**g**) and a correct prediction (green, **b**,**d**,**f**,**h**) from similarity bins 0–20 (**a**,**b**), 30–40 (**c**,**d**), 40–60 (**e**,**f**) and 80–90 (**g**,**h**).

Figure 5 shows some examples of successful and failed predictions from different similarity bins along with the ground truth complex, and the closest training system found based on the SuCOS–pocket similarity score. As the similarity to the training systems increases, we observe, as expected, a growing overlap in terms of molecular shape and common substituents between the target ligand and the one found in the closest training system. However, in some cases, important chemical features are overlooked during inference, as exemplified in Fig. 5e. Despite being explicitly defined in the input SMILES (Simplified Molecular Input Line Entry System), all the methods place the 5-chlorobenzofuran-3-aminomethyl group with incorrect chirality, perhaps mimicking the 5-chloro-2-methoxyphenyl group found in the closest training system. Regarding proteins, globally, we observe that their structures and binding pockets are accurately predicted across all similarity bins, as depicted in Fig. 4d. This is not always the case, Fig. 5c shows an example in which the protein is also mispredicted: the ground truth is a protein kinase in inactive Asp-Phe-Gly (DFG)-out conformation^38^; however, the predicted structure adopts a DFG-in conformation. Although Fig. 5 provides some useful visual cues on when these current methods are likely to perform well, it also paints a clear picture of their inherent limitations in generating accurate predictions for drug-like molecules beyond the training data.

### Room for improvement in sampling and ranking of ligand poses

**Fig. 6:**
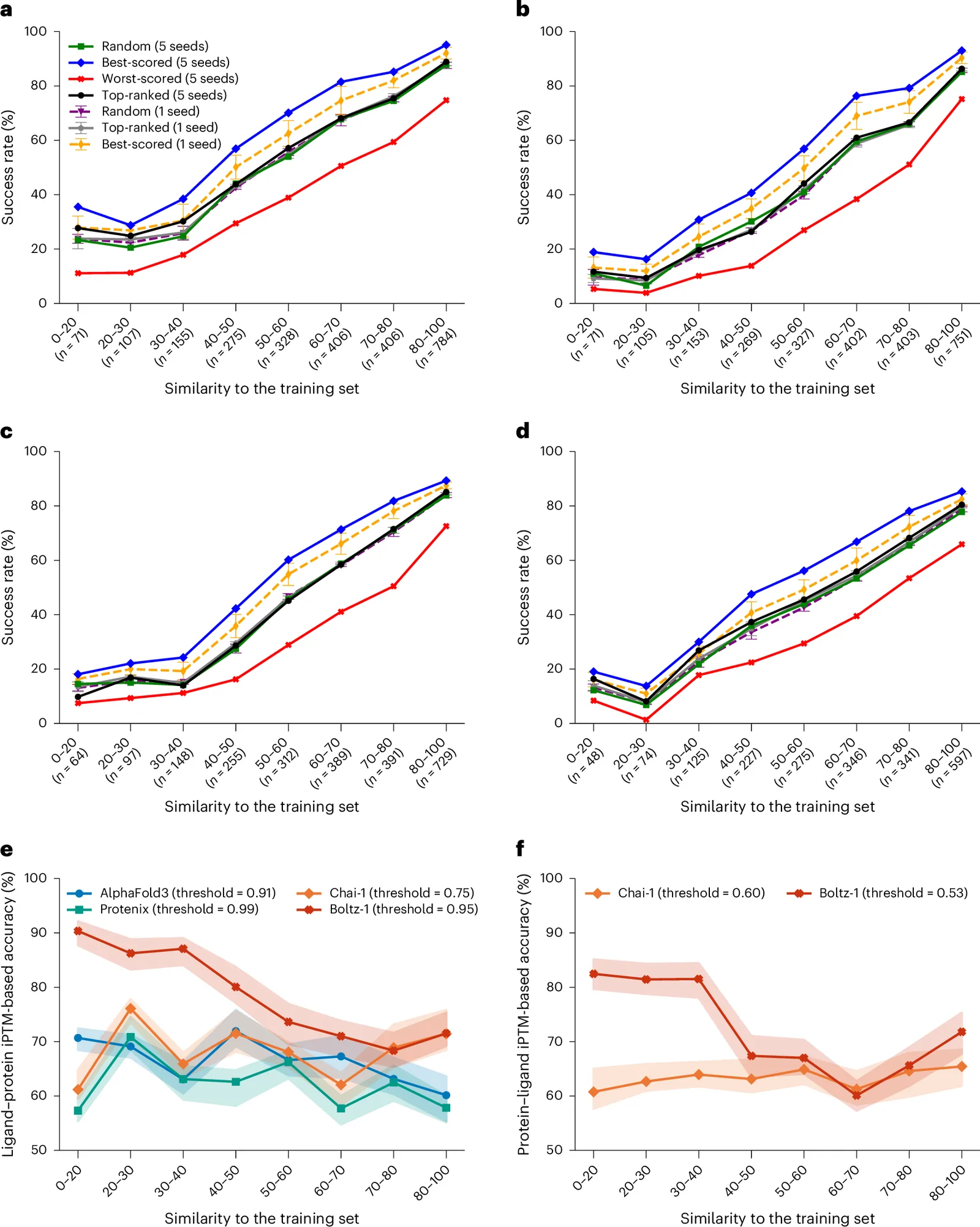
Confidence metrics in and across systems. **a**–**d**, Success rate across similarity bins for a randomly selected model across 25 (green squares), the best-scored model (blue diamonds, best LDDT-PLI across 25), worst-scored model (red crosses, worst LDDT-PLI across 25), top-ranked model (black circles, with the highest ranking_score), top-ranked across 1 seed (gray pentagons, with standard deviation as whiskers across the 5 seeds), randomly selected from 1 seed (purple triangles) and best-scored across 1 seed (yellow diamonds). **a**, AlphaFold3. **b**, Protenix. **c**, Chai-1. **d**, Boltz-1. **e**, Ligand–protein chain-pair iPTM-based success classification accuracy for the four methods. In total, 250 positive and 250 negative systems were sampled for each similarity bin and each method, with 1,000 bootstrap estimates shown as shaded areas. **f**, As **e** but using protein–ligand chain-pair iPTM only for Chai-1 and Boltz-1.

To de-correlate the effect of predicting poses versus ranking them, we look at the accuracies for all 25 models (5 seeds, 5 diffusion samples) for each system across the four methods. Figure 6a–d depicts, for each method and SuCOS–pocket similarity bin, the success rates of the best-scored (closest to the ground truth), worst-scored (furthest from the ground truth), a randomly picked model from the 25 and the top-ranked model selected based on each method’s ranking scores. We also look at the distribution of top-ranked, best-scored and randomly picked models across the five seeds, to demonstrate the impact of using multiple seeds. Although the top-ranked model is better than the worst-scored model generated (black versus red), there is still a gap to the best possible model (in blue) and it is usually not much better than random selection (in green). In addition, we observe that using multiple seeds is currently not useful at inference time for any of the methods. Although more-accurate ligand poses are sampled with five seeds (blue versus yellow), the ranking picks similar models when using one or five seeds (as seen by the black line overlapping with the gray and purple lines). Nevertheless, sampling the correct pose continues to be the main bottleneck in improving the accuracy, since even the best-scored poses do not flatten the curve.

We also investigated the usability of the ligand–protein chain-pair iPTM (interface predicted template modelling) score for differentiating accurate and inaccurate poses across predicted models of different systems. We calculate this score as the average of chain-pair iPTM scores across all protein chains to the considered ligand chain. For each method, we independently determine the best threshold as the optimal operating point of the receiver operating characteristic (ROC) curve across all predicted systems, where positives are predicted models with LDDT-PLI >0.8 and r.m.s.d. <2 Å compared to the reference, and negatives are predicted models that do not satisfy these criteria. Thus, this chosen iPTM threshold has the best trade-off between sensitivity and specificity. The optimal threshold is different for each method, with Protenix being the most optimistic (0.99) about its predictions, Chai-1 being the least optimistic (0.75), and AlphaFold3 and Boltz-1 having thresholds of 0.92 and 0.95 respectively. Thus, previously established template modelling (TM) score thresholds cannot be used for model selection, and the confidence scores are not comparable across the four methods.

Figure 6e shows the accuracy across the similarity bins on classifying predicted models as positives or negatives based on the determined iPTM threshold for each method. Equal numbers of positive and negative cases (250 each) were chosen for every bin, to counteract the imbalance across the similarity bins. Overall, the iPTM-based accuracy does not seem to correlate with the training set similarity. Of the four assessed methods, Boltz-1 shows a clear advantage over the others in accurately classifying their predictions with 70–90% accuracy across the similarity bins, whereas the other methods stay below 75%. Compared to the other methods, Boltz-1 introduced several architectural and training modifications to their confidence model, clearly reflected in the improved iPTM-based ranking power.

An additional peculiarity differentiating the four methods is the asymmetrical nature of the chain-pair iPTM matrix for Boltz-1 and Chai-1, where the protein–ligand chain-pair iPTM has a different value from the ligand–protein chain-pair iPTM. This is not the case for AlphaFold3 and Protenix, both of which have symmetrical matrices due to averaging across protein and ligand chains. The protein–ligand chain-pair iPTM shows lower ranking power (Fig. 6f).

### Toward realistic physics baselines

An obvious question arising from our analysis is the performance of physics-based rigid ligand-docking methods on Runs N’ Poses. Unfortunately, comparing rigid docking to cofolding is not straightforward for several reasons. First, docking requires an input protein structure while cofolding starts from the protein sequence. Second, the input protein conformation is required to be very close to the ground truth *holo* conformation of the protein, with even slight side chain deviations often resulting in very poor performance^18^. Third, docking benefits greatly from a pocket definition, where the center of the pocket and a rough box size describing the area in which the ligand should fit is prespecified. Without this information (also known as blind docking), performance is expected to be quite poor^39^. Finally, the scoring aspect of docking in which predicted poses are ranked to find one top pose is known to be unreliable^40^ and thus typical docking benchmarks use the best of *n* poses to disentangle the impact of pose prediction versus scoring. With all of these caveats in mind, we designed five baselines serving different purposes (Table 1, methodological details are described in the Methods).

**Table 1:** Five noncofolding baselines and their respective inputs, methods, assumptions and aims

Name	Input protein conformation	Pocket specification	Docking method	Idealized scenarios	Aim
Redocking	GT receptor	GT ligand	GLIDE-SP^62^	Existence of co-crystal complex of the input protein and ligand	Demonstrate the overall physical plausibility of the ground truth poses
AF3-dock	Top-ranked AF3-cofolded receptor	AF3-cofolded ligand	GLIDE-SP	—	Demonstrate the combined pocket location and conformation prediction capabilities of AF3
AF3-dock-ideal	Best-scored AF3-cofolded receptor	GT ligand	GLIDE-SP	Good model selector, knowledge of pocket location and size	Demonstrate the pocket conformation prediction capabilities of AF3
HM-dock-ideal	Closest training system HM receptor (with SWISS-MODEL^63^)	GT ligand	GLIDE-SP	Good template selector, knowledge of pocket location and size	Demonstrate whether a good pocket conformation is present in the training data
HM-transplant	Closest training system HM receptor	Template ligand	MCS-constrained RDKit conformer aligned to template ligand with AutoDock Vina^64^ local optimization	Good template selector	Demonstrate how far one can get using a naive training point transfer approach

All five have the additional assumption of having a good pose selector that can rank the best of 25 predicted poses. The closest training system is defined as the one having the highest SuCOS–pocket similarity to the ground truth system. AF3, AlphaFold3; GT, ground truth; HM, homology model.

**Fig. 7:**
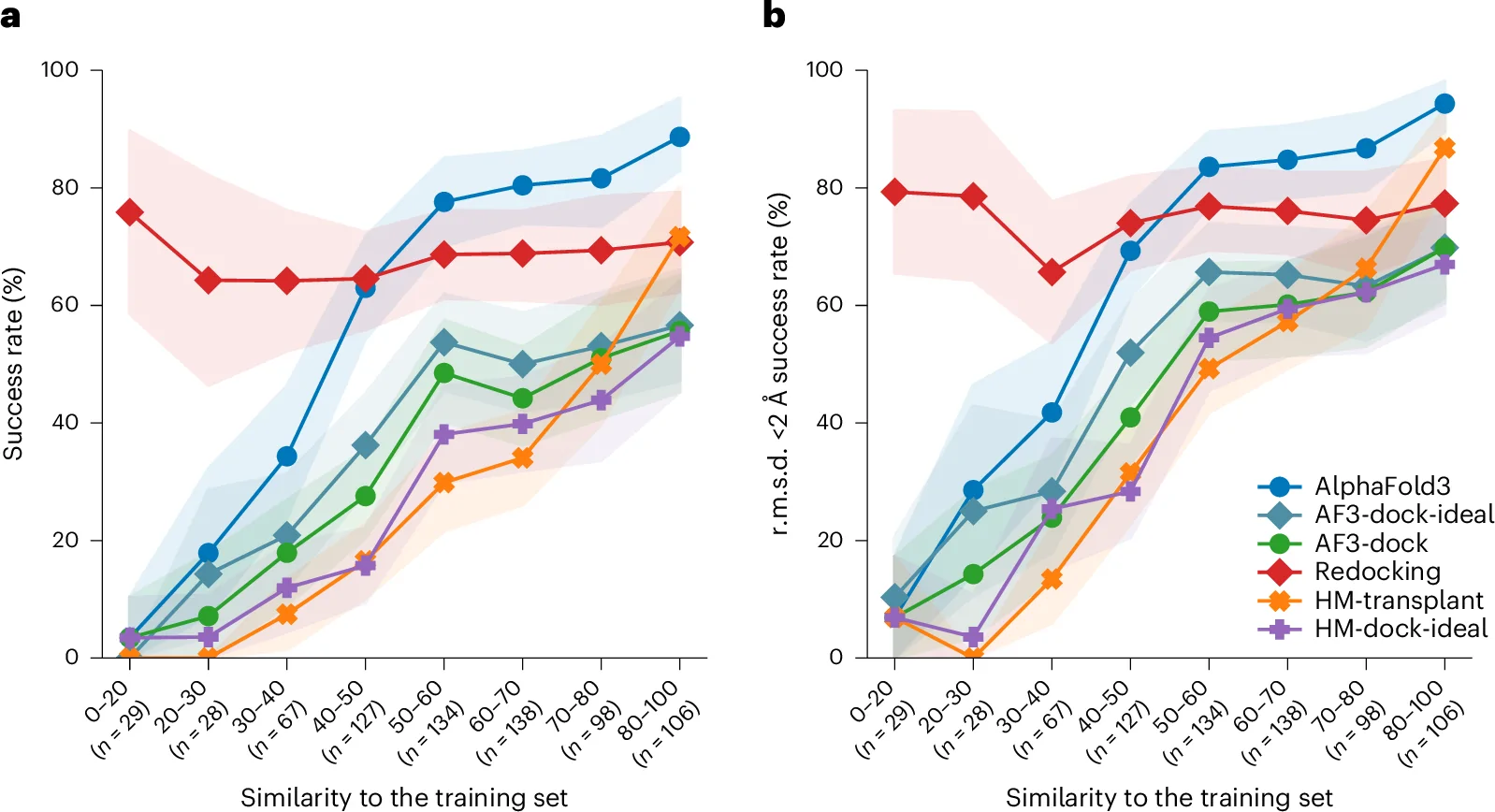
Prediction accuracy versus training set similarity for AlphaFold3 versus docking and template baselines. **a**,**b**, Success rate, defined as the percentage of system ligands with <2 Å r.m.s.d. and >0.8 LDDT-PLI (**a**) or with <2 Å r.m.s.d. alone (**b**) for all common subset system ligands in each similarity bin across AlphaFold3 and the methods described in Table 1. Shaded regions correspond to the 95% confidence interval, calculated from 1,000 bootstrap samples for each bin and method.

Figure 7 shows the results of these baselines in comparison to AlphaFold3 (best of 25 models), on a subset of Runs N’ Poses consisting of single-protein, single-ligand systems with only distinct ligands. As shown in Table 3, docking methods typically failed to produce outputs at rates higher than AlphaFold3 and more along the lines of Boltz-1. HM-Transplant and HM-dock-ideal have more missing systems in the lower bins because of a lack of good homology models in these bins. AF3-dock-ideal has more missing systems than AF3-dock in the lower bins likely due to the specification of a pocket that is not prepared to bind a ligand in the former case. To have enough statistical power in the lower bins to interpret results, we show results on the common subset of 727 systems between AlphaFold3 and Redocking, and set the missing systems of other baselines as 0 LDDT-PLI and 100 Å r.m.s.d.

The Redocking results demonstrate that protein–ligand complexes in our dataset behave more or less equally with physics-based scoring functions when the receptor protein conformation and pocket are known. Thus, there is nothing particularly difficult about the cases in the lower similarity bins from a physics perspective. However, redocking is an unrealistic, idealized scenario because the receptor protein conformation in a ligand-bound form is not available without the ground truth complex. The more realistic scenario of AF3-dock instead uses the AlphaFold3-cofolded receptor (best of 25) and docks the ligand to the same pocket where AlphaFold3 cofolded the ligand. Here we see again the dependence on similarity to the training set, but also that in higher bins the performance of just using AlphaFold3 is not reached. Note that this uses rigid docking, thus demonstrating that the pockets predicted by AlphaFold3 are not in the correct conformation and/or location for docking the ligand in the lower bins. We also show AF3-dock-ideal, which uses the ground truth pocket location, to demonstrate that although pocket prediction is a relevant and important aspect of docking and using the ground truth pocket improves results in cases in which the AlphaFold3 pocket is in the wrong location (green versus teal), the main bottleneck is still the protein and pocket conformation. We found that using the top-scored or best-ranked AlphaFold3 receptor did not make a recognizable difference in results, confirming that pocket location makes the difference between the AF3-dock (in green) and AF3-dock-ideal (in teal) performances.

Our two homology modeling-based baselines aim to further highlight the existence of memorization: the homology model built from the closest training system based on SuCOS–pocket similarity (HM-dock-ideal) performs similarly as input to docking as AF3-dock-ideal, indicating that the main bottleneck is the template selection step. Most strikingly, the very simple approach of transplanting the ligand using the template ligand to constrain the pose (HM-transplant) performs quite close to AlphaFold3 in the higher bins and similar to the docking baselines in the lower bins, especially when using only an r.m.s.d. threshold of <2 Å to define the success rate (Fig. 7b). This was also seen in CASP16 where consensus template docking methods often reached AlphaFold3 performance on select systems, sometimes even by combining information from multiple templates^41^.

When including the LDDT-PLI threshold (Fig. 7a), success rates in the higher bins drop for docking and template transplant baselines because the local optimizations performed to avoid clashes in imperfect pockets lead to protein–ligand distances that do not pass the stringent LDDT-PLI >0.8 threshold. Here we note that specific metrics such as PLI fingerprint recovery^42^ could be used to see more granular results but often require careful complex preparation steps that are difficult to automate across such hybrid methods.

Note that of the five baselines benchmarked, only AF3-dock can actually be used in a real-world setting (still with the need of a good pose selector) because all of the others have heavy assumptions on protein and pocket prediction. Thus, we do not aim for this analysis to be a head-to-head comparison of cofolding and docking because too many intermediate variables affect the performance of docking and making an ideal workflow that addresses all of these variables is beyond the scope of this work. For example, a flexible approach that allows protein side chain movements for induced fit docking could also improve performance on lower bins but often requires careful selection of flexible residues and ranking, and is thus difficult to automate. In general, an experienced computational chemist would likely be able to reach closer to redocking level of performance across the bins on a case-by-case basis, by paying close attention to receptor preparation, flexible residue handling, pocket prediction and pose selection. Recent approaches using machine learning are also being developed to address this, showing promising results including on the lower similarity bins of our set^43^.

## Discussion

Overall, our benchmark shows that the current generation of deep learning cofolding methods still has a long way to go to accurately model unseen protein–ligand complexes. We demonstrate that the performance of current approaches strongly correlates with the similarity to their training data, regardless of the metric used to define success or the subsets considered.

Our analysis suggests that both sampling and ranking contribute to the limitations of these methods. In cases in which the prediction was not accurate, the correct pose is either not sampled at all or, when it is, the method fails to select it during ranking, further highlighting the need for improvement in both aspects.

The results on prevalent ligands found in multiple training systems across diverse pockets indicate that data scarcity remains a major challenge for these methods. Given the vastness of chemical space, we are far from the optimal data regime required for deep learning models to generalize effectively. Data augmentation strategies such as cross-docking^44,45^, direct usage of underlying electron densities^46–48^ or using small-molecule data in isolation^49,50^ may help improve the diversity and coverage of training data across chemical space, as well as across different pairs of pockets and ligands. In addition, a large number of experimentally resolved protein–ligand complexes, binding assays useful for cross-docking and negative binding data are available in pharmaceutical companies and closed-source settings. A collective effort to make such industrial data available for training, perhaps through infrastructures for distributed privacy-preserving federated learning such as the MELLODDY project^51^, or large-scale targeted experimental structure determination as planned by the OpenBind consortium^52^, could greatly improve the data scarcity situation.

Even with increased dataset diversity, it remains unclear whether the optimal architecture has been developed for this task or if current methods would fully exploit these advancements. For example, the generalizability of Boltz-2 did not discernibly improve compared to Boltz-1, despite being trained on more data (Fig. 2). Drawing parallels with the protein structure prediction task, AlphaFold2^21^ benefited from coevolutionary signals extracted from billions of sequenced proteins to learn an approximate energy landscape^53,54^ and truly achieved generalizable performance also on difficult proteins without homologous structures in the PDB, with the caveat of having poorer performance on orphan proteins without such coevolutionary information^55^. All-atom cofolding methods do not currently rely on any such external nonstructural data for encoding and representing ligand interactions. Incorporating physics-based terms to more accurately model PLIs, potentially from simulations, conformational ensembles or other sources, is likely needed to achieve more exciting results in this field. Our results are also in line with recent work demonstrating, through adversarial examples, significant divergences from expected physical behaviors in protein–ligand predictions^56^. This highlights the need to integrate robust physical and chemical priors to enhance method generalization, also shown by our docking baselines and others^43^. Overall, we believe that both increased dataset diversity and more appropriate architectures are required to see progress in the field.

All in all, our findings stress the importance of specialized benchmark datasets and evaluation techniques tailored to deep learning PLI prediction. We observed that most released high-quality complexes have a high similarity to the training set (Fig. 1; 75% system ligands have >50 SuCOS–pocket similarity), demonstrating that difficult test systems consisting of novel molecules or binding pockets, both highly relevant to drug discovery and design, are underrepresented in general, and the commonly used time split approaches would not capture these. In fact, 87% of the complexes in the commonly used PoseBusters benchmark dataset^20^ have a SuCOS–pocket similarity >50 to the training set, explaining the high performance scores typically obtained by these methods on this set. Although it is easy to conclude that cofolding methods can in general be trusted in cases in which similar complexes have been seen in the PDB, our failure mode examples demonstrate that caution is required even in this scenario, because small changes in ligand chemistry can lead to changes in poses that cofolding methods would likely not capture, and instead would confidently return previously seen results. This was also seen in concurrent work^56^ where mutations in the binding pocket, which should disrupt ligand binding, were ignored. However, we find that applying physics-based docking methods for ligand placement also requires care because the results are heavily dependent on the accuracy of the protein and pocket conformation used.

Of the four benchmarked methods, only the authors of Protenix^27^ list memorization artifacts as an expected limitation, further underscoring the difficulty in uncovering such biases without extensive benchmarking combining measures of prediction performance with measures of data leakage. Since its first release, Runs N’ Poses data and similarity metrics have been used in recent benchmarking efforts (for example CAMEO^57^, PoseBench^58^), and also by method developers to investigate the biases in their models (for example, DragonFold^59^, OpenFold3^60^, PEARL^61^).

We demonstrate the pitfalls of commonly used similarity thresholds such as 40% sequence identity, proving them to be insufficient to assess method generalization. Although SuCOS similarity proved most relevant for this analysis because it combines both volume and chemical features of the small molecule, it would also be interesting to disentangle these two aspects and inspect how much these methods make use of simple volume overlap over chemical similarity. In addition, in cases in which there are multiple training systems containing the same protein and ligand but with the ligand binding in different conformations, it would be interesting to know whether these methods choose one pose to output or if they combine substructures from different poses. By providing all the prediction files and similarity scores against the entire training set, we hope to enable such analyses and drive more granular interpretation of method performance. The results also show that the physical plausibility of generated poses is sometimes suboptimal, again precluding use in real-world drug-discovery campaigns. Promisingly, the diffusion steering strategy implemented in Boltz-1x largely solves these issues, and could straightforwardly be implemented into other cofolding methods using a similar architecture. However, this does not change the generalization trend, still calling for concerted improvements in architecture and training to address memorization.

We note that the only requirement to apply the Runs N’ Poses benchmark in its entirety on a new method is a structural training cutoff of 30 September 2021, with enough data also available to assess later cutoffs (Fig. 2). Because the dataset was constructed in an automated fashion, there may be a few complexes that do not qualify as biologically relevant. We expect that careful inspection and continued use will reveal such cases, but because we demonstrate the robustness of the trends discussed across various stratifications, we do not expect this to alter the conclusions.

The future of benchmarking for deep learning methods, as we move toward more complex multidimensional tasks such as cofolding, requires different measures to assess leakage and difficulty, whether for protein–protein, protein–ligand or nucleic acid-containing complexes. This is also particularly crucial for tasks with even more limited data, such as those involving covalent bonds and modified residues. In this context, we look forward to input from the community on how to best support innovation in this field and strive toward more-accurate methods, along with fairer ways of assessing their capabilities.

## Methods

### Dataset curation

We ran the PLINDER ingestion pipeline^30^ on all entries in the PDB^19^ released after 30 September 2021 up to 9 January 2025 to obtain PLINDER systems, defined as all chains from a bioassembly within 6 Å of a ligand chain. The resulting systems were further filtered to pass the following criteria:

- Solved by X-ray crystallography
- From the first bioassembly
- With at most five ligand chains defined in the system and at most five protein chains in the bioassembly
- No systems with covalent ligands
- No systems with all ligands marked as cofactors or oligopeptides or nucleotides or saccharides
- Passing the following X-ray validation criteria (as defined for the PLINDER benchmark dataset) to ensure that they are reliable ground truth for evaluation:
  - Resolution ≤3.5 Å
  - R-factor ≤0.4
  - *R*_free_ (free R-factor) ≤ 0.45
  - *R* − *R*_free_ ≤0.05
  - Coordinates exist for all heavy atoms in the ligand and pocket
  - No alternative configurations in the ligand and pocket
  - No clash outliers for the ligand
  - No crystal contacts detected for the ligand
- Passing the following statistics criteria to ensure that they are relevant ligand interactions:
  - Having between 3 and 50 detected PLIP^65^ interactions
  - Having between 5 and 100 protein residues within 6 Å of the ligands in the system
  - The biggest ligand in the system having a molecular weight between 200 and 800.

Systems passing these filters were further clustered to 80% sequence identity, and the system redundancy cluster was defined as the combination of the sequence cluster and the set of CCD^66^ codes of the ‘proper’ ligands in the system; that is, ligands not labeled as ions or artifacts (as defined in PLINDER^30^). Only one system per redundancy cluster was chosen, the one with the maximum number of PLIP^65^ interactions detected.

These filtering steps resulted in 2,600 systems used as inputs for the four prediction methods (Table 2). For systems in which there were polymer chains present in the bioassembly that were not part of the system definition, we used all protein chains in the bioassembly and all ligand chains part of any PLINDER system involving that bioassembly as input for the four methods. Although all ligands in the pocket were used for modeling (including ions), the accuracy metrics of only proper ligands were analyzed, only for the ligand and protein chains defined for the ground truth PLINDER system, taking the best-scored when multiple mappings were found. Results are presented per proper ligand and no aggregation was done for multi-ligand systems, resulting in 3,047 proper ligands across all the analyzed systems. Twenty-one systems contained nucleic acid chains close to the ligand pocket. Although cofolding models can handle such input, the chains are currently excluded from PLINDER system definitions and thus would not be scored as part of the system. Future PLINDER releases with fixed system definitions for nucleic acid chains will solve this issue but because of the scarce numbers we chose to drop these 21 systems for the presented analysis. A further 72 system ligands from 63 systems in the common subset could not be analyzed because they could not be read with RDKit, and thus similarity scores could not be obtained. For some systems, the SMILES for the ligand in the PDB does not contain chiral information while the ground truth has at least one chiral center; that is, the molecule given as input to each cofolding method may not be the molecule expected as the output. This is not a straightforward issue to fix in an automated fashion because the ground truth may not have all the atoms resolved and so cannot be the source of truth for extracting the input SMILES. Although technically in Runs N’ Poses we ensured that all atoms are resolved, this is not true for many PDB entries, indicating that these methods are also trained on such cases where the chirality information in the input does not match the output.

**Table 2:** Counts of the full Runs N’ Poses dataset and the obtained predictions from the methods benchmarked

Method	PDB IDs	Systems	All ligands	Proper ligands	Multi-ligand systems	Multi-protein systems
Post 30 September 2021						
Total	2,585	2,600	4,282	3,047	401	790
AF3	2,396	2,409	3,860	2,710	296	738
Protenix	2,310	2,323	3,726	2,609	281	702
Chai-1	2,280	2,292	3,683	2,572	275	685
Boltz-1	2,126	2,139	3,372	2,400	256	626
AF3-NT	2,398	2,411	3,861	2,711	295	738
Boltz-1x	2,078	2,091	3,322	2,338	242	608
RFAA	1,759	1,769	2,718	1,946	173	507
Post 1 June 2023						
Total	1,025	1,028	1,685	1,187	142	415
Boltz-1	841	844	1,322	933	87	347
Boltz-2	868	871	1,396	961	88	361

AF3-NT, AlphaFold3 run without templates; RFAA, RoseTTAFold All-Atom.

To define prevalent ligands, the number of training systems with ligand analogs was calculated for each system by counting systems before the training cutoff containing ligands with >0.9 ligand topological fingerprint Tanimoto similarity^35^.

### Running all-atom prediction methods

Following the protocol of the PoseBusters benchmark conducted by the authors of AlphaFold3^23^, AlphaFold3 (v.3.0.0), Boltz-1 (v.0.4.1), Boltz-1x (v.1.0.0), Boltz-2 (v.2.0.3) Chai-1 (v.0.5.1) and Protenix (v.0.3.4) were run on one A100 40 graphics processing unit each with 10 recycling steps, 200 diffusion sampling steps, 5 seeds and 5 diffusion samples per seed, which resulted in a total of 25 models per system for each method. We use the ligand–protein chain-pair iPTM score for ranking the 25 models, although using the default ranking score showed very similar results.

The input files were prepared according to the instructions for each method. Scripts for input file preparation can be found in our associated GitHub repository. To ensure that the prediction outcomes had no reliance on the depth or quality of the multiple sequence alignments (MSAs) generated by different pipelines, we used the same MSAs as input for all methods. The standard AlphaFold3 MSA generation pipeline was run to obtain the non_paired and paired MSAs for each system. The same MSAs were given to Protenix in.a3m format with the pairing and non_pairing options, with pairing_db set to uniprot as this is the format AlphaFold3 uses for their paired MSA. For Chai-1, both paired and nonpaired a3m files were used to generate the aligned.pqt with the provided a3m-to-pqt function, with taxonomy identifiers extracted from UniProt for the paired MSA. The same pairing keys were used to generate the custom MSA CSV files for the Boltz methods. For unpaired sequences, pairing keys were set to −1. Thus, each method uses the same MSAs and pairing strategies as input. In addition, Chai-1 predictions were made with the evolutionary scale modeling (ESM) embedding option enabled and AlphaFold3 predictions were made with templates enabled. AlphaFold3 was also run without templates for comparison (AF3-NT). For RoseTTAFold All-Atom, we followed the standard procedure as described in its documentation.

We did not manage to obtain prediction results from all methods across all 2,600 systems in the dataset. Some systems (128) failed to produce output across all four methods owing to: (1) incorrect specification of modified amino acids, (2) reaching memory limits in A100 40G graphics processing units, and (3) failures in the conformer generation step. Table 2 details the number of systems that could be modeled by all the different methods, with Boltz-1 or Boltz-1x having the highest number of missing predictions. In total we had 229,887 predictions across the four methods used for common subset analysis, namely AlphaFold3 (with templates), Boltz-1, Chai-1 and Protenix, resulting in a common subset of 2,311 proper ligands (3,288 including ions) across 2,077 systems. Boltz-2 was only run on 1,028 systems released after its training cutoff of 1 June 2023.

### Running docking baselines

We developed four docking and one template baseline benchmarked in the section ‘Toward realistic physics baselines’ on all single-ligand, single-protein systems with distinct ligands. An overview of these baselines can be found in Table 1.

The four docking baselines use GLIDE-SP (Schrödinger Release 2025-3)^62^ on a given input protein PDB file and grid (pocket) specification set to return 25 poses and remaining default parameters. For each protein–ligand pair, the ligand is prepared from SMILES using LigPrep as ligprep -epik -pht 1 -s 16 -ismi ligand.smiles -omae ligands.maegz, and the protein is prepared from an input receptor PDB file (baseline-specific) using PrepWizard as prepwizard receptor.pdb receptor.mae -rehtreat. The grid is defined by an input ligand conformer (baseline-specific), using the arithmetic mean of the ligand coordinates as the center of the grid and the outer box size defined by the radius of gyration of the ligand conformer, as described in ref. ^67^. The inner box size is set to 10. Finally, GLIDE-SP is run with POSES_PER_LIG and POSTDOCK_NPOSE both set to 25, with the confgen docking method.

For the Redocking baseline, the input receptor PDB file is obtained from the ground truth PLINDER system and the ligand conformer used for grid definition is obtained from ground truth ligand standard data file (SDF). Both AF3-dock and AF3-dock-ideal use the protein chain from the best-scored AlphaFold3 model as the input receptor PDB file. AF3-dock uses the AlphaFold3-predicted ligand conformer to define the grid, while AF3-dock-ideal uses the ground truth ligand SDF file with the AlphaFold3 protein superposed to the ground truth system to ensure the ligand is in the correct frame.

For HM-dock-ideal and HM-transplant we used SWISS-MODEL^63^ to obtain homology models for each system, giving the template as the training system with the highest SuCOS–pocket similarity. For HM-dock-ideal the homology modeled protein, superposed to the ground truth system, is used as the input receptor PDB file, and the grid is defined by the ground truth ligand conformer.

The HM-transplant baseline does not make use of GLIDE. Instead, the following protocol is run for each system. First, the ETKDGv3 forcefield^68^ is used with RDKit^33^ to generate 25 conformers from the input ligand SMILES. Second, the maximum common substructure (MCS) is found in the template ligand (that is, obtained from the PDB used as template for making the homology model) compared to the input ligand SMILES, with RDKit’s rdFMCS.FindMCS function. Third, each of the 25 generated conformers are superposed to the MCS molecule and then minimized with the universal force field^69^ with distance constraints added for each matching atom, followed by a second minimization using the Merck molecular force field^70^ with the same distance restraints. Fourth, these template-constrained conformers are then placed into the homology modeled protein aligned to the template ligand. Fifth, an AutoDock Vina^64^ local optimization step is then run on the 25 protein–ligand complexes to fix clashes. Apart from the homology modeling template selection step, none of these steps assume any knowledge of the ground truth complex.

All 5 baselines return 25 ligand poses for each complex, from which the one with the best LDDT-PLI score is retained. Table 3 details the number of systems that could be modeled by the different baselines, along with the percentage of failures across different similarity bins.

**Table 3:** Counts of modeled systems for docking baselines

Method	Total	0–40 (% missing)	40–70 (% missing)	70–100 (% missing)
AlphaFold3	874	2.5	1.5	1.2
Redocking	753	18.2	15.5	19.1
AF3-dock	733	22.1	16.9	22.7
AF3-dock-ideal	724	27.3	16.9	21.5
HM-dock-ideal	691	34.4	18.8	21.1
HM-transplant	810	19.5	7.9	3.9
Total count	907	154	478	256

The first column lists the number of systems modeled by each method, while the remaining columns list the percentage of missing systems (of the totals in the last row) for SuCOS–pocket similarity bins of 0–40, 40–70 and 70–100.

### Accuracy scoring

To evaluate the accuracy of predicted models, we use the compare-ligand-structures action of OpenStructure^34^ (v.2.8.0), which returns the binding-site superposed symmetry-corrected ligand r.m.s.d. (hereafter referred as just r.m.s.d.), the LDDT-LP and the LDDT-PLI. For LDDT-PLI calculation we enable the ‘added model contacts’ flag, which further penalizes protein–ligand contacts present in the model that are not in the reference (note that this is the default for LDDT-PLI since OpenStructure v.2.9). In addition, we calculated the *F*_1_ score of pocket recovery, in which true positives are defined as pocket residues found in both the predicted and ground truth structures (where pocket residues are defined as those having any atom within 6 Å of any ligand atom). All of these scores are calculated per ligand using the receptor CIF (crystallographic information file) and ligand SDF files saved by PLINDER as the ground truth for each system. Only the scores for ligands labeled as proper ligands (excluding ions and artifacts) were analyzed, and multi-ligand systems form multiple data points in our analysis, one per proper ligand. We provide code to extract relevant scores from the OpenStructure output JSON files in our associated GitHub repository.

Because we are working with cofolding methods, in which both the protein pocket and ligand pose have to be accurately modeled, we redefine the commonly used ‘Success rate’ measure as the fraction of systems with <2 Å r.m.s.d. and >0.8 LDDT-PLI. Supplementary Fig. 4a shows the distribution of r.m.s.d. and LDDT-PLI values across all predictions. This addresses two types of outliers on the top right and the bottom left.

The top right outlier section in the Fig. 4a contains 1.6% of predictions with LDDT-PLI >0.8 but r.m.s.d. >2 Å, sometimes consisting of cases such as the example shown in Supplementary Fig. 4b. While the global superposition here would have resulted in a low r.m.s.d., and the LDDT-PLI is high (0.86) because of many of the protein–ligand distances with one domain being maintained, the binding-site superposed r.m.s.d. is high (3.72 Å) because the two binding-site residues shown in sticks are used to perform the superposition and the top domain is incorrectly modeled. This area also has some cases in which a part of the ligand not interacting with the protein is flexible and therefore its orientation is not biologically relevant or well-resolved. Although r.m.s.d. might correct the over-optimistic results of LDDT-PLI in the former case, in the latter case LDDT-PLI might be a more meaningful measure on its own. However, because <2% of cases across all 229,887 predicted models fall into this area and we cannot automatically differentiate between the two cases, we opted to treat these as failures.

The bottom left outlier section in the Fig. 4a contains 8.3% of the predictions with r.m.s.d. <2 Å but LDDT-PLI <0.8, consisting of cases in which the pose is correct but the pocket is wrongly modeled. The example in Supplementary Fig. 4c shows the global superposition of the two complexes, which would lead to a very low r.m.s.d. value. Although the binding-site superposed r.m.s.d. shows worse results (1.63 Å), it would still be considered a success if using only r.m.s.d. <2 Å as the success criteria. LDDT-PLI, however, is 0.43, correctly reflecting that the PLIs are not maintained.

To measure the physical plausibility of the predicted poses, we ran the suite of checks implemented in PoseBusters^20^ using the ‘redock’ configuration on all predicted models, and report the number of systems that pass all default checks.

### Similarity scoring

PLINDER calculates 14 metrics covering protein sequence (similarity and identity), protein structure (Local Distance Difference Test on Cα atoms; CA-LDDT); pocket coverage, identity and structure; and the similarity of PLIP interactions^30^. In addition, the SuCOS, which measures the overlap of volume and chemical features between two ligand poses, has been shown to better differentiate binding pose similarity than r.m.s.d. and PLI-based scores^32^. Thus, we calculated three more metrics working in concert with the protein-based scores.

Because SuCOS calculation requires superposition between ligand poses, we align the ligand poses of a query and target system using RDKit’s rdShapeAlign.AlignMol^33^ command. The superposed molecules were then used to calculate the SuCOS score^31^, as implemented in https://github.com/susanhleung/SuCOS. These three scores are further multiplied by the pocket coverage (pocket_qcov, divided by 100 to get the fraction), which measures the percentage of residues within 6 Å of the ligand in the query system that align with residues within 6 Å of the ligand in the target system, to obtain relevant scores measuring the similarity of ligand poses in the same pocket. Although approaches combining protein and pocket-level superposition were attempted, they turned out to be too sensitive to the efficacy of rigid protein superposition.

The main similarity score used in this paper to define training set similarity, find the closest training system as well as cluster similar systems, unless otherwise specified, is the SuCOS multiplied by the pocket_qcov (SuCOS–pocket). To obtain these values for each system in our set, we perform a Foldseek^71^ search against the entire PDB and calculate similarities between any pair of test and train systems where any protein chain has an alignment. Note that pocket_qcov does not directly consider residue identity or similarity, and just checks that the residues are aligned.

Despite our best efforts, we cannot be sure that we are always able to accurately calculate similarity to the training set, because our methods rely on a combination of alignment and superposition that may fail in edge cases. Also, we only search for similarity against PLI systems, excluding artifact-only systems and those with oligopeptides, oligosaccharides or oligonucleotides >10 in length, thus potentially missing cases of training set proteins complexed with oligo, protein or nucleotide chains that resemble small-molecule binding. However, this latter similarity would indicate transfer of binding modes from different modalities and perhaps may not be considered memorization.

Using PLINDER’s pli_qcov scores, which count the fraction of shared PLI types detected by PLIP^65^ on aligning residues, gave similar results (Supplementary Fig. 3l); however, because cofolding methods are not trained to recapitulate interactions, we wanted to account for cases in which the SuCOS similarity of the ligand pose remains high but pocket residue conformational changes result in different PLIs being detected (low pli_qcov similarity, high SuCOS–pocket similarity), as well as cases in which the part of the ligand interacting with the protein remains the same, but other regions of the ligand change in conformation (high pli_qcov similarity, low SuCOS–pocket similarity).

The dataset was clustered using community clustering on the graph connecting systems having SuCOS–pocket similarity scores >50. Cluster representatives are chosen as those with the lowest similarity to the training set.

Supplementary Fig. 5 shows examples of the types of ligands found in each training set similarity bin.

### Reporting summary

Further information on research design is available in the Nature Portfolio Reporting Summary linked to this article.

## Online content

Any methods, additional references, Nature Portfolio reporting summaries, source data, extended data, supplementary information, acknowledgements, peer review information; details of author contributions and competing interests; and statements of data and code availability are available at https://doi.org/10.1038/s41594-026-01797-5.

## Supplementary information

Supplementary Information Supplementary Figs. 1–5 and Table 1.

Reporting Summary

Peer Review File

## Data Availability

The dataset for benchmarking use is available via Zenodo at https://doi.org/10.5281/zenodo.14794785 (ref. ^72^), including the sequences, SMILES and MSAs used as input for each prediction, and the ground truth CIF and SDF files for each system. For each method, we provide the prediction CIF files, CSV files containing the prediction accuracies (ligand r.m.s.d., LDDT-PLI, pocket backbone r.m.s.d. and LDDT-LP), and confidence scores (ranking score, ligand–protein and protein–ligand chain-pair iPTM) for all 25 models across all systems. Further, we provide a CSV file annotating systems with all similarity metrics to the training system with the highest SuCOS–pocket similarity, and the similarity bin that the system falls into, for the September 2021 training cutoff. We also provide a Parquet file with all the similarities found to every system in the PDB up to 9 January 2025, enabling assessment of later training cutoffs. An ML-ready version of the benchmark can be found via Polaris Hub^73^ at https://polarishub.io/benchmarks/plinder-org/runs-n-poses. All predicted models along with associated metadata will be made available in ModelArchive^74^ with the project identifier ma-rnp.

## Code availability

Scripts to recreate the figures in this manuscript, prepare inputs for the different methods and evaluate predictions are available via GitHub at github.com/plinder-org/runs-n-poses.
