# Supplementary material for: Evaluating generalization in protein–ligand cofolding methods
Source: Nat Struct Mol Biol. 2026 May 8;33(5):782–94. doi: 10.1038/s41594-026-01797-5 (PMC13661253; doi:10.1038/s41594-026-01797-5)
Supplement: Supplementary file 1 [file 41594_2026_1797_MOESM1_ESM.pdf]

nature structural & molecular biology

Supplementary information https://doi.org/10.1038/s41594-026-01797-5

Evaluating generalization in protein–ligand cofolding methods

In the format provided by the authors and unedited

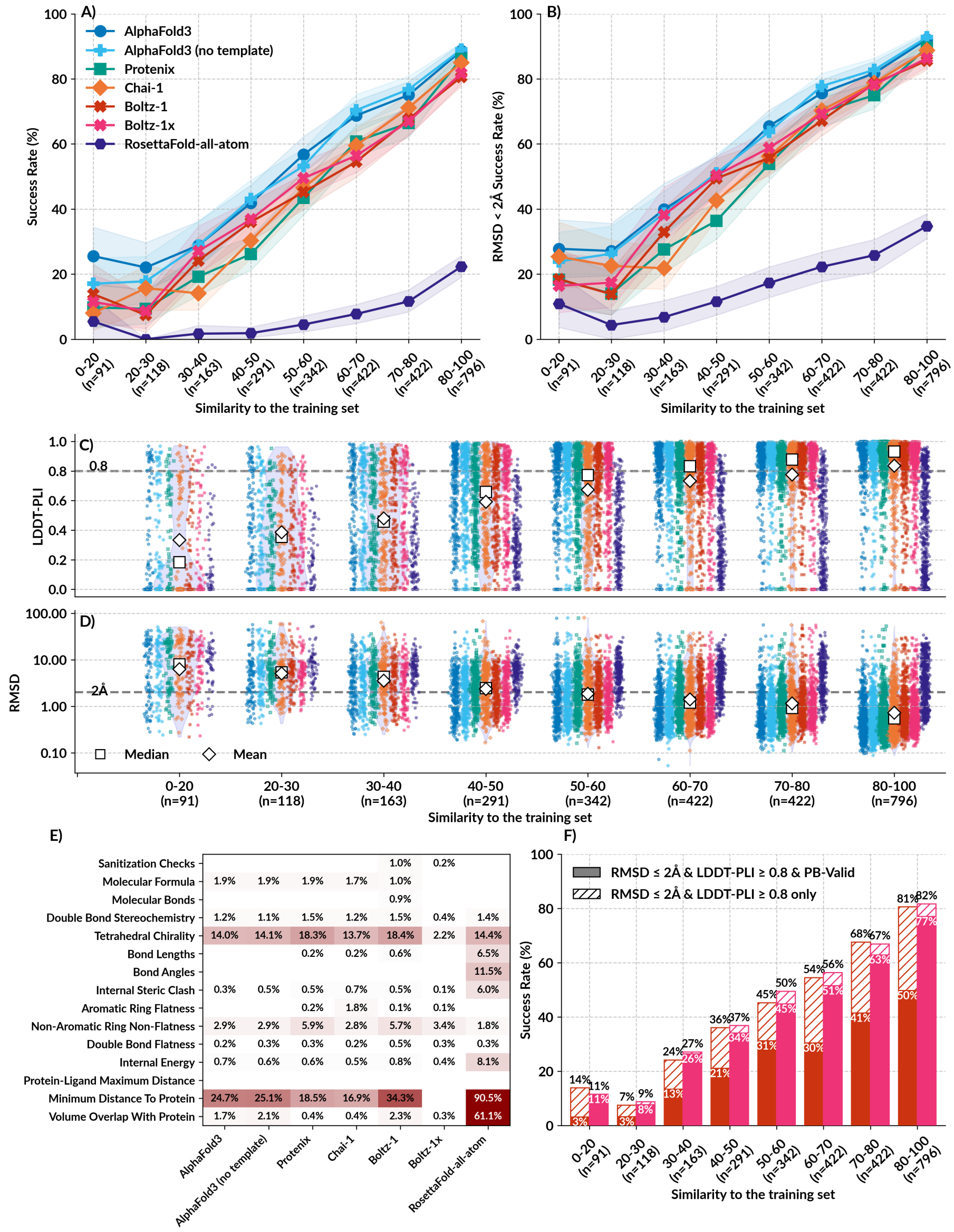

Supplementary Figure S1 Prediction accuracy vs. training set similarity across all predicted models. A) The success rate, defined as the percentage of system ligands with <2Å RMSD and >0.8 LDDT-PLI, for all system ligands in each similarity bin across the assessed methods. Shaded regions correspond to the 95% confidence interval, calculated from 1,000 bootstrap samples for each bin and method. **B)** same as A but with success rate defined only by <2Å RMSD. **C), D)** The distribution of LDDT-PLI and RMSD values respectively for all methods displayed as a violin plot, with each individual method being shown as coloured scatter points. **E)** The percentage of systems from each assessed method (columns) failing each listed PoseBusters physical plausibility check (rows). **F)** Same as panel A with added physical validity checks for Boltz-1 and Boltz-1x. The striped bars show the share of predictions of each method that have <2Å RMSD and >0.8 LDDT-PLI and the solid bars show the subset that in addition have valid geometries and energies, i.e., pass all PoseBusters tests and are therefore 'PB-Valid'.

Index	Cluster Size	PDB ID	Description	Similarity bins
1	171	7EN9	Crystal structure of SARS-CoV-2 3CLpro in complex with the non-covalent inhibitor WU-02	80-100 (79), 70-80 (46), 60-70 (25), 50-60 (15), 40-50 (6)
2	136	8OKU	Salt-Inducible Kinase 3 in complex with an inhibitor	60-70 (38), 70-80 (37), 80-100 (28), 50-60 (19), 40-50 (11), 30-40 (3)
3	100	5SH0	Crystal structure of human phosphodiesterase 10 in complex with IZT	50-60 (33), 40-50 (26), 30-40 (16), 60-70 (13), 80-100 (5), 20-30 (4), 70-80 (3)
4	92	8HUU	Crystal structure of HCoV-NL63 main protease with S217622	80-100 (43), 60-70 (20), 70-80 (20), 40-50 (5), 50-60 (4)
5	73	7S3Z	Structure of rat neuronal nitric oxide synthase heme domain in complex with V0P	80-100 (51), 70-80 (16), 60-70 (5), 50-60 (1)
6	43	8JOO	Crystal structure of cytochrome P450 IkaD from Streptomyces sp. ZJ306, in complex with the substrate ikarugamycin	80-100 (23), 60-70 (5), 70-80 (5), 30-40 (4), 40-50 (3), 50-60 (3)
7	35	7G00	Crystal structure of human FABP1 in complex with WII	60-70 (13), 50-60 (10), 40-50 (6), 70-80 (3), 80-100 (2), 30-40 (1)
8	28	7UBO	Crystal structure of the first bromodomain of human BRDT in complex with the inhibitor CCD-956	70-80 (7), 80-100 (7), 50-60 (6), 60-70 (5), 40-50 (2), 30-40 (1)
9	28	7S1S	PRMT5/MEP50 crystal structure with MTA and MRTX-1719 bound	80-100 (12), 40-50 (6), 50-60 (3), 60-70 (3), 30-40 (2), 70-80 (2)
10	26	7V0H	Crystal structure of putative glucose 1-dehydrogenase from *Burkholderia cenocepacia* in complex with NADP and a potential reaction product	80-100 (13), 60-70 (7), 70-80 (6)

Supplementary Table S1 Representatives of populated clusters. Sizes of the ten most populated clusters, along with the PDB ID and description of the cluster representative with the lowest training set similarity for each, and distribution of the cluster across different training SuCOS-pocket similarity bins.

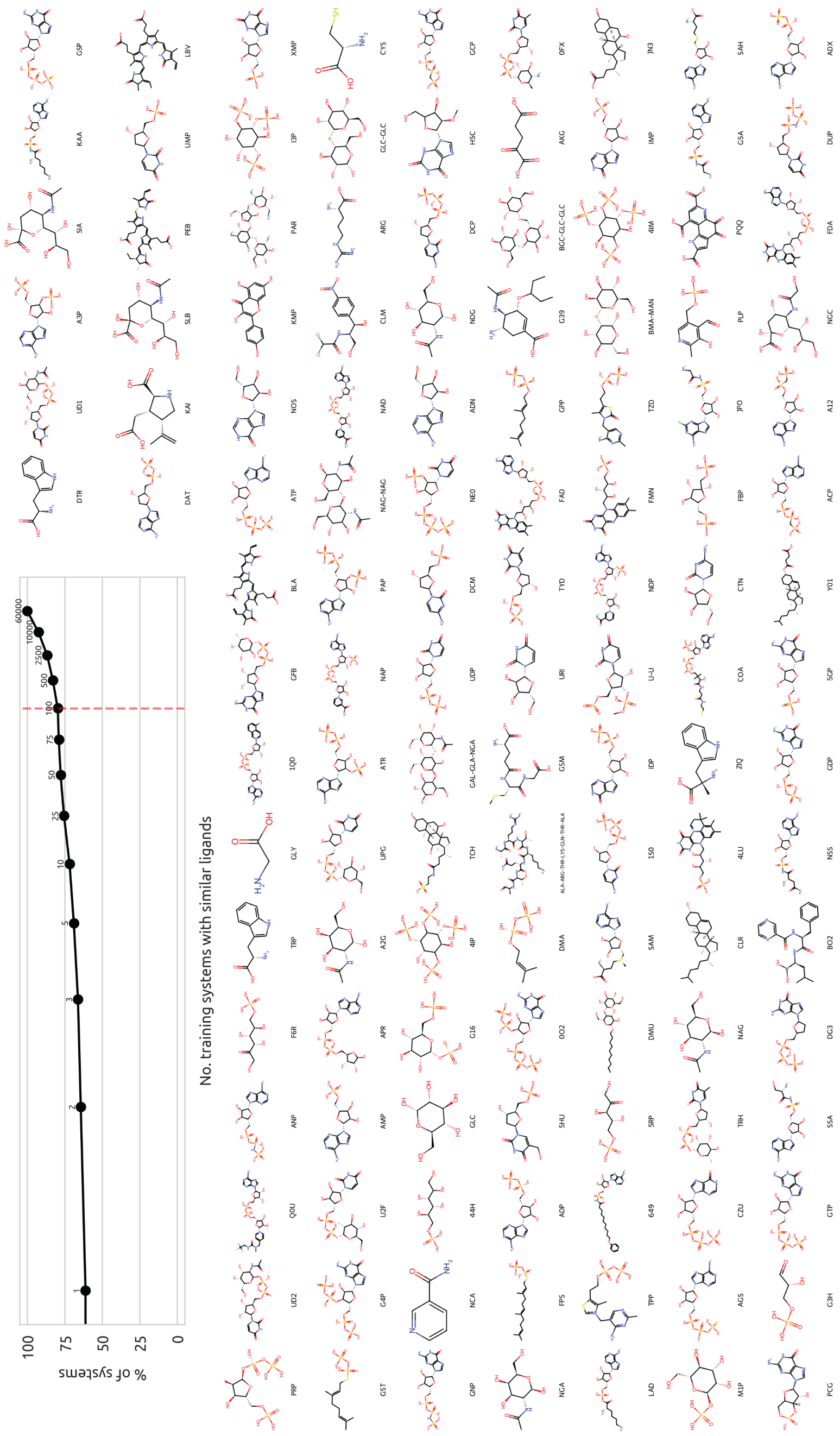

Supplementary Figure S2 Prevalent ligands. The percentage of system ligands (Y-axis) having different numbers of training systems with analogous ligands (X-axis, log scale), and all the ligands within systems with >100 analogous ligand training systems.

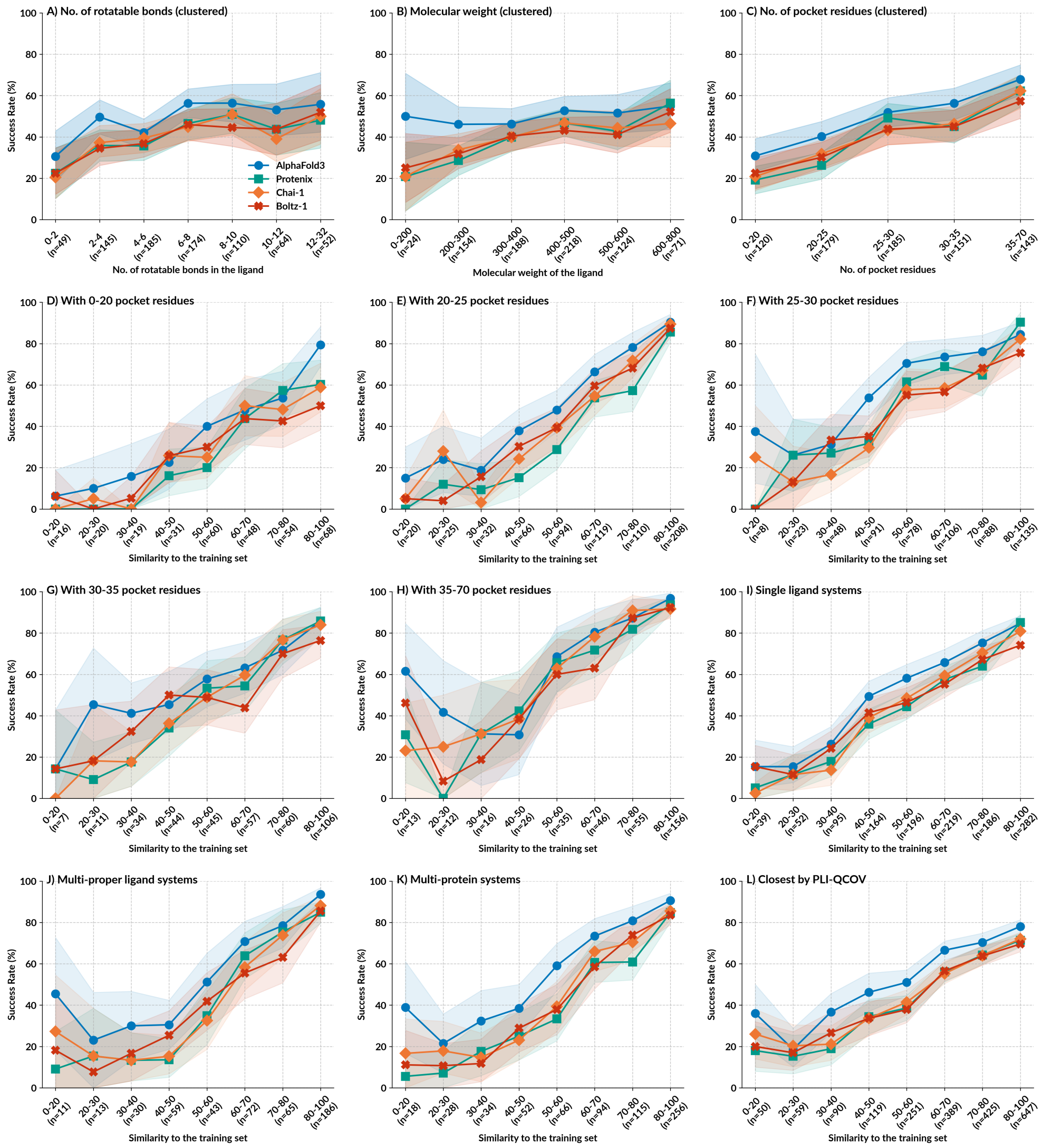

Supplementary Figure S3 Success rate distributions for different stratifications. Success rates for the different methods and the best model of the four, **A)** for clustered system ligands with differing numbers of rotatable bonds. **B)** for clustered system ligands with differing molecular weights. **C)** for clustered system ligands with differing numbers of residues in the binding pocket. **D-H)** or system ligands having binding pockets (residues within 6 Å of the ligand) defined by D) 0-20, E) 20-25, F) 25-30, G) 30-35, and H) >35 residues respectively. **I-K)** across the different SuCOS-pocket similarity bins only for systems having I) a single ligand, J) multiple proper ligands, and K) multiple protein chains, respectively. **L)** across different `pli_qcov` similarity bins when using `pli_qcov` similarity to find the closest training system.

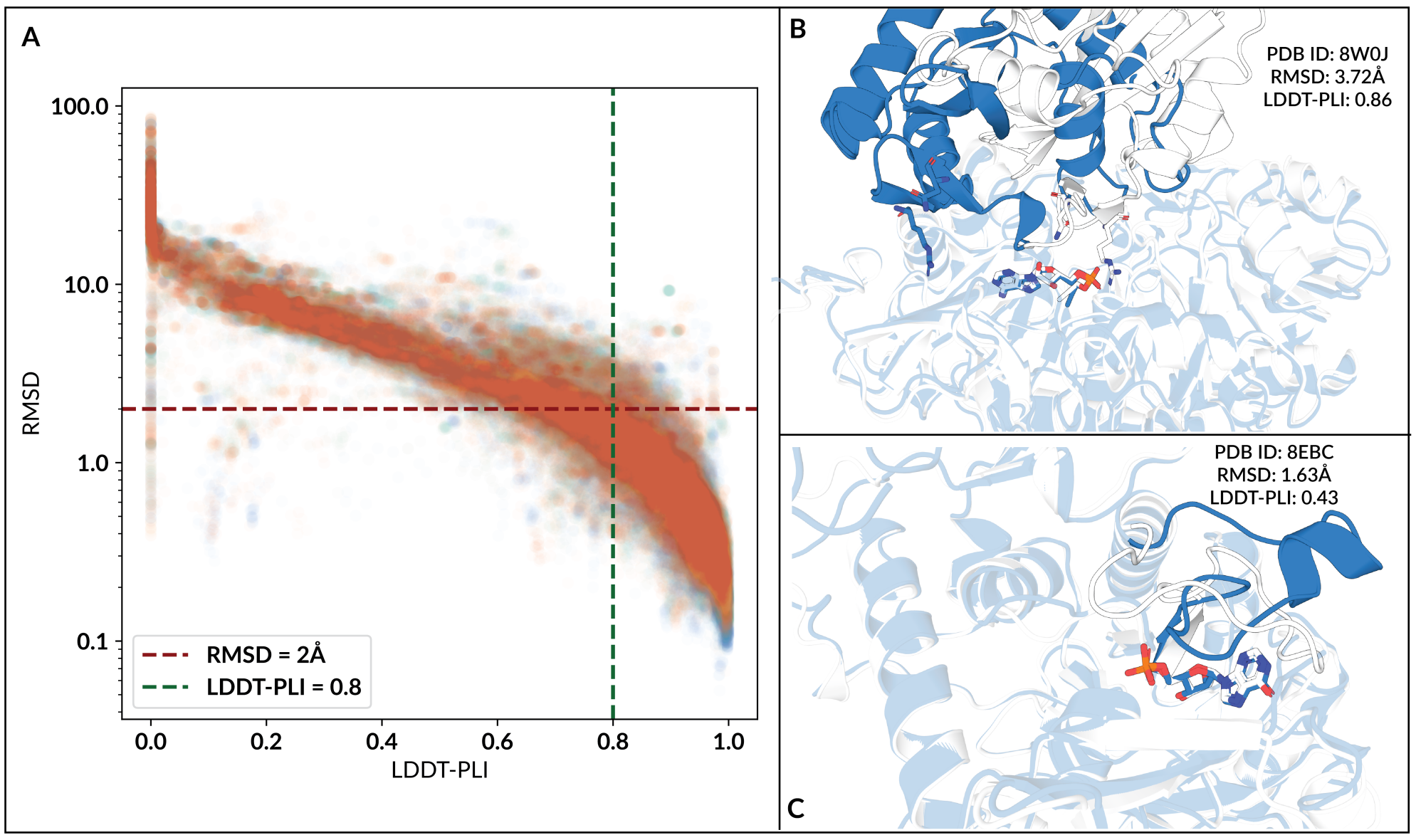

Supplementary Figure S4 LDDT-PLI and RMSD scores across all predictions. A) The distribution of binding site superposed RMSD (log-scale) and LDDT-PLI for all 229,887 predictions with 2Å RMSD and 0.8 LDDT-PLI shown as dashed lines. **B)** An example with high RMSD and high LDDT-PLI, the binding site residues used for performing the superposition are shown as sticks in the predicted structure (blue) and ground truth (gray). **C)** An example with low RMSD and low LDDT-PLI, prediction in blue and ground truth in gray.

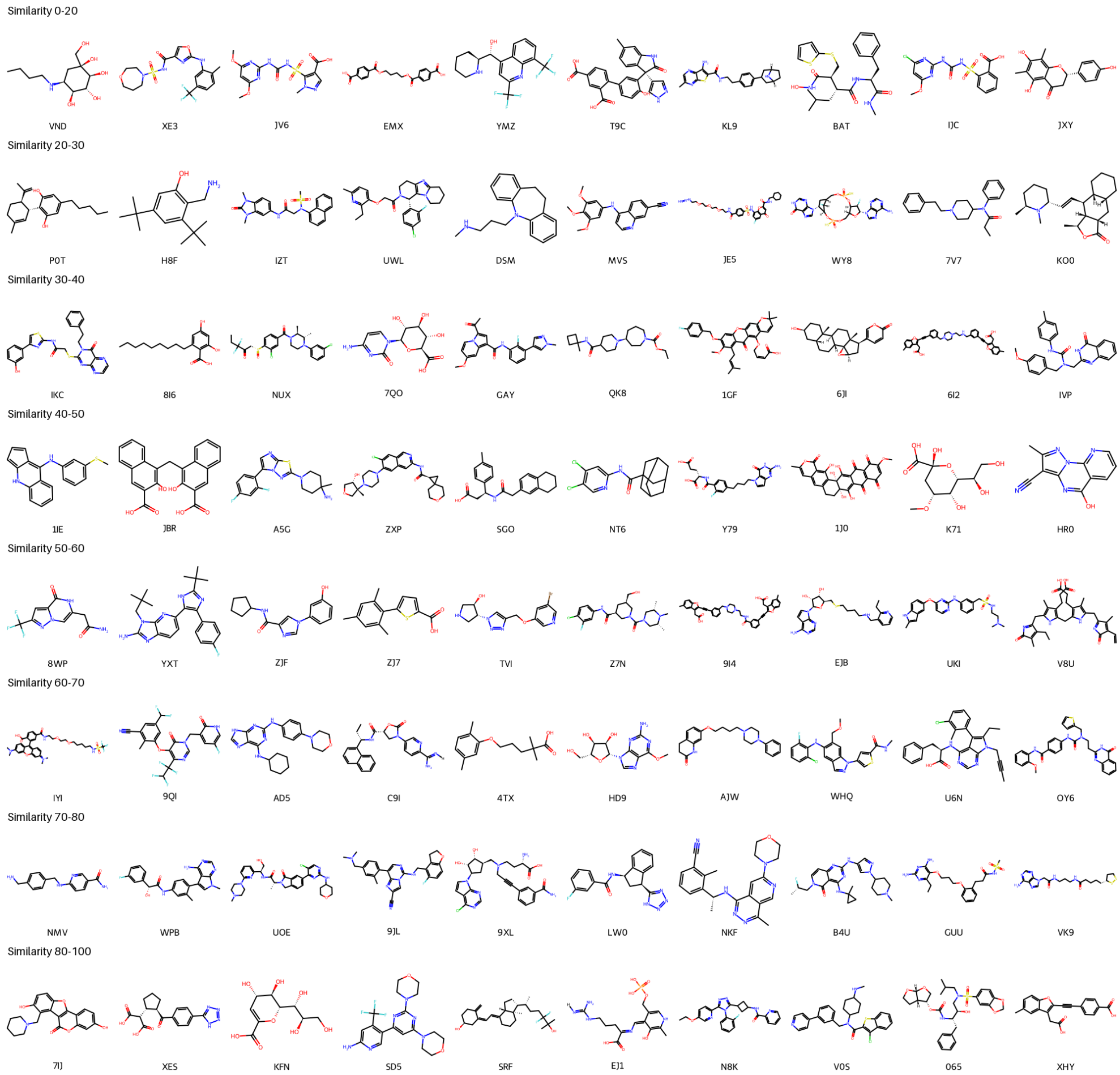

Supplementary Figure S5 Ten examples of small molecules found in each training set similarity bin.
