# Supplementary material for: Evaluating generalization in protein–ligand cofolding methods
Source: Nat Struct Mol Biol. 2026 May 8;33(5):782–94. doi: 10.1038/s41594-026-01797-5 (PMC13661253; doi:10.1038/s41594-026-01797-5)
Supplement: Supplementary file 2 [file 41594_2026_1797_MOESM2_ESM.pdf]

nature portfolio

Corresponding author(s): Janani Durairaj

Last updated by author(s): Mar 3, 2026

Reporting Summary

Nature Portfolio wishes to improve the reproducibility of the work that we publish. This form provides structure for consistency and transparency in reporting. For further information on Nature Portfolio policies, see our Editorial Policies and the Editorial Policy Checklist.

Statistics

For all statistical analyses, confirm that the following items are present in the figure legend, table legend, main text, or Methods section.

n/a	Confirmed	
☐	☒	The exact sample size (*n*) for each experimental group/condition, given as a discrete number and unit of measurement
☐	☒	A statement on whether measurements were taken from distinct samples or whether the same sample was measured repeatedly
☒	☐	The statistical test(s) used AND whether they are one- or two-sided<br>*Only common tests should be described solely by name; describe more complex techniques in the Methods section.*
☐	☒	A description of all covariates tested
☐	☒	A description of any assumptions or corrections, such as tests of normality and adjustment for multiple comparisons
☐	☒	A full description of the statistical parameters including central tendency (e.g. means) or other basic estimates (e.g. regression coefficient) AND variation (e.g. standard deviation) or associated estimates of uncertainty (e.g. confidence intervals)
☒	☐	For null hypothesis testing, the test statistic (e.g. *F*, *t*, *r*) with confidence intervals, effect sizes, degrees of freedom and *P* value noted<br>*Give P values as exact values whenever suitable.*
☒	☐	For Bayesian analysis, information on the choice of priors and Markov chain Monte Carlo settings
☐	☒	For hierarchical and complex designs, identification of the appropriate level for tests and full reporting of outcomes
☒	☐	Estimates of effect sizes (e.g. Cohen's *d*, Pearson's *r*), indicating how they were calculated

Our web collection on statistics for biologists contains articles on many of the points above.

Software and code

Policy information about availability of computer code

Data collection	- PLINDER - https://github.com/plinder-org/plinder/tree/custom_cif (commit b01598ba99d606c7c7f503aa1ad7507110ea977d) used for filtering and selecting the dataset, collecting the ground truth CIF and SDF files, and annotating complexes with metadata from the PDB.<br>- AlphaFold3 (v3.0.0), Boltz-1 (v0.4.1), Boltz-1x (v1.0.0), Boltz-2 (v2.0.3), Chai-1 (v0.5.1), Protenix (v0.3.4), RosettaFold-All-Atom (commit fe4cefafc1467093565ed7af7d250e9c2f4a620a) were used to run cofolding and the resulting predicted models (CIF files) and associated metadata (e.g confidence scores) were collected. SWISS-MODEL, GLIDE-SP (Schrödinger Release 2025-3) , and AutoDock Vina (v1.2.5) were used to perform the docking baselines.
Data analysis	- All code used to generate the figures and scripts for modelling and analysis along with example outputs are available at https://github.com/plinder-org/runs-n-poses<br>- PLINDER - https://github.com/plinder-org/plinder/tree/custom_cif (commit b01598ba99d606c7c7f503aa1ad7507110ea977d), PLIP (v2.3.0), Foldseek (9.427df8a) and RDKit (2024.09.6) and code adapted from https://github.com/susanhleung/SuCOS were used to calculate similarity scores.<br>- OpenStructure (v2.8.0) was used to calculate accuracy metrics (LDDT-PLI, RMSD etc.) for all predicted models.<br>- PoseBusters (v0.4.4) was used to calculate ligand physical plausibility checks.<br>- Matplotlib (v3.9.2) and PyMol (v2.5-master-d24468af) were used to generate all the figures.

For manuscripts utilizing custom algorithms or software that are central to the research but not yet described in published literature, software must be made available to editors and reviewers. We strongly encourage code deposition in a community repository (e.g. GitHub). See the Nature Portfolio guidelines for submitting code & software for further information.

Data

Policy information about availability of data

All manuscripts must include a data availability statement. This statement should provide the following information, where applicable:

- Accession codes, unique identifiers, or web links for publicly available datasets
- A description of any restrictions on data availability
- For clinical datasets or third party data, please ensure that the statement adheres to our policy

We provide the entire dataset for benchmarking use at doi.org/10.5281/zenodo.14794785, including the sequences, SMILES, and MSAs used as input for each prediction, and the ground truth CIF and SDF files for each system. For each method, we provide the prediction CIF files, CSV files containing the prediction accuracies (ligand RMSD, LDDT-PLI, pocket backbone RMSD, and LDDT-LP), and confidence scores (ranking score, ligand-protein and protein-ligand chain pair iPTM) for all 25 models across all systems. For the AlphaFold3 models, the Output Terms of Use is also provided. Further, we provide a CSV file annotating systems with all similarity metrics to the training system with the highest SuCOS-pocket similarity, and the similarity bin that the system falls into, for the September 2021 training cutoff. We also provide a Parquet file with all the similarities found to every system in the PDB up to 9 January 2025, enabling assessment of later training cutoffs. Scripts to recreate the figures in this manuscript, prepare inputs for the different methods, and evaluate predictions can be found at github.com/plinder-org/runs-n-poses. An ML-ready version of the benchmark can be found on Polaris Hub at https://polarishub.io/benchmarks/plinder-org/runs-n-poses. All predicted models along with associated metadata will be made available in ModelArchive with the project identifier ma-rnp.

Research involving human participants, their data, or biological material

Policy information about studies with human participants or human data. See also policy information about sex, gender (identity/presentation), and sexual orientation and race, ethnicity and racism.

Reporting on sex and gender	N/A
Reporting on race, ethnicity, or other socially relevant groupings	N/A
Population characteristics	N/A
Recruitment	N/A
Ethics oversight	N/A

Note that full information on the approval of the study protocol must also be provided in the manuscript.

Field-specific reporting

Please select the one below that is the best fit for your research. If you are not sure, read the appropriate sections before making your selection.

☒ Life sciences ☐ Behavioural & social sciences ☐ Ecological, evolutionary & environmental sciences

For a reference copy of the document with all sections, see nature.com/documents/nr-reporting-summary-flat.pdf

Life sciences study design

All studies must disclose on these points even when the disclosure is negative.

Sample size	Sample size was determined by selected complexes after the training cutoff of deep learning methods and binning them based on similarity to the training set.
Data exclusions	The following numbers of systems were excluded from the analysis:<br>- 21 systems contained nucleic acid chains close to the ligand pocket.<br>- 72 system ligands from 63 systems in the common subset could not be analysed as they could not be read with RDKit, and thus similarity scores could not be obtained.<br>- 128 systems failed to produce output across all four methods due to (1) incorrect specification of modified amino acids, (2) reaching memory limits in A100 40G GPUs, and (3) failures in the conformer generation step.
Replication	We obtained 25 predicted models with 5 different seeds per system per cofolding method to enable assessment of variability across samples. For the docking baselines we allowed 25 poses to be generated for each system. We performed bootstrapping in all plots to show confidence intervals.
Randomization	Systems for the dataset were selected from the PDB using cutoff date and various other filters as well as redundancy sampling. We measured impact of different covariates (e.g pocket size, number of proteins, number of ligands etc.) by showing results on different stratifications of the data.

Blinding

Blinding is not relevant to our study as the computational methods are run with preconfigured settings and analysed with automated computational software both of which do not vary by human factors

Reporting for specific materials, systems and methods

We require information from authors about some types of materials, experimental systems and methods used in many studies. Here, indicate whether each material, system or method listed is relevant to your study. If you are not sure if a list item applies to your research, read the appropriate section before selecting a response.

Materials & experimental systems

n/a	Involved in the study
☒	☐ Antibodies
☒	☐ Eukaryotic cell lines
☒	☐ Palaeontology and archaeology
☒	☐ Animals and other organisms
☒	☐ Clinical data
☒	☐ Dual use research of concern
☒	☐ Plants

Methods

n/a	Involved in the study
☒	☐ ChIP-seq
☒	☐ Flow cytometry
☒	☐ MRI-based neuroimaging

Plants

Seed stocks

N/A

Novel plant genotypes

N/A

Authentication

N/A
