# Supplementary material for: Evaluating generalization in protein–ligand cofolding methods
Source: Nat Struct Mol Biol. 2026 May 8;33(5):782–94. doi: 10.1038/s41594-026-01797-5 (PMC13661253; doi:10.1038/s41594-026-01797-5)
Supplement: Supplementary file 3 [file 41594_2026_1797_MOESM3_ESM.pdf]

nature portfolio

Peer Review File

Evaluating generalisation in protein-ligand cofolding methods

Corresponding Author: Dr Janani Durairaj

Version 0:

Decision Letter:

31st Oct 2025

Dear Dr. Durairaj,

Thank you again for submitting your manuscript "Have protein-ligand cofolding methods moved beyond memorisation?" to the journal. I apologize for the delay in responding, which resulted from the difficulty in obtaining referee reports. We now have comments (below) from the 3 reviewers who evaluated your paper. In light of those reports, we remain interested in your study and would like to see your response to the comments of the referees in the form of a revised manuscript.

You will see that while the reviewers value the insights provided by this work, Reviewer #1 requests to comment on whether the co-folding methods work differently on single-ligand vs multi-ligand systems, and to contrast findings to a recent independent study on protein-ligand pose prediction methods. Reviewer #2 requests to filter out incorrect geometries in order to report truer success rates of co-folding models in drug design applications, to conduct a systematic analysis of ligand geometries to quantify the misassigned stereochemical configurations and other types of chemical misrepresentations occurring in the generated ligand structures, and to adopt a more cautious interpretation of the “Best” curve to clearly distinguishing between complementarity and result accumulation effects. Reviewer #3 Requests adding comparison with physics-based docking methods to determine whether the poor performance of co-folding methods is a direct result of memorization or inherent difficulty of SuCOS-pocket systems, and additional analysis to contextualize SuCOS-pocket scores with core accuracy metrics and physical validity checks.

Please be sure to address/respond to all concerns of the referees in full in a point-by-point response and highlight all changes in the revised manuscript text file. We expect to see your revised manuscript within 6 weeks. If you cannot send it within this time, please don't hesitate to contact us to discuss an extension; we will still consider your revision provided that no similar work has been accepted for publication at NSMB or published elsewhere. We are committed to providing a fair and constructive peer-review process; please do not hesitate to contact us if you would like to discuss the reviews or anticipate any delay with the resubmission.

REPORTING AND DATA AVAILABILITY: Please provide updated files as follows with your revision. As you already know, we put great emphasis on ensuring that the methods and statistics reported in our papers are correct and accurate.

1. REPORTING SUMMARY: Please provide an updated file:

https://www.nature.com/documents/nr-reporting-summary.pdf

This form is a dynamic ‘smart pdf’ and must be downloaded and completed in Adobe Reader.

2. REPORTING OF STRUCTURAL DATA: If there are additional or modified structures in the revision, please submit the corresponding PDB validation reports with your other files, and use the figshare integration system to provide access to maps, half-maps, and models.

Manuscripts reporting new structures should contain a table summarizing structural and refinement statistics. Please include these tables for new cryo-EM or -ET (https://www.nature.com/documents/nr-tables-cryo-em.doc and modifying this file for ET), NMR (https://www.nature.com/documents/nr-tables-nmr.doc) and X-ray crystallography (https://www.nature.com/documents/nr-tables-xray.doc). To facilitate assessment of the quality of the structural data, a stereo image of a portion of the electron density map (for crystallography papers) or of the superimposed lowest energy structures (>10; for NMR papers) should be provided with the submitted manuscript. If the reported structure represents a novel overall fold, a stereo image of the entire structure (as a backbone trace) should also be provided. For cryo-EM structures, a

representative micrograph showing individual particles should be provided in the figures alongside a processing workflow (please see our Editorial for more information: https://www.nature.com/articles/s41594-025-01567-9).

DATA DEPOSITION: We require deposition of coordinates (and, in the case of crystal structures, structure factors) into the Protein Data Bank with the designation of immediate release upon publication (HPUB). Electron microscopy-derived density maps and coordinate data must be deposited in EMDB and released upon publication. Deposition and immediate release of NMR chemical shift assignments are requested. To avoid delays in publication, dataset accession numbers must be supplied with the revised manuscript and appropriate release dates must be indicated at the galley proof stage.

3. REPORTING OF LIGHT MICROSCOPY DATA: For any revision that includes light microscopy data, we ask our authors to please include a completed light microscopy reporting table [https://www.nature.com/documents/Light_microscopy_reporting_table.xlsx] to ensure the methods are described thoroughly. The table will be available to reviewers and

ultimately published, should the manuscript be accepted at the journal. When submitting the revised version of your manuscript, please pay close attention to our href="https://www.nature.com/nature-portfolio/editorial-policies/image-integrity">Digital Image Integrity Guidelines.

4. CODE AND COMPUTATIONAL WORK: If newly developed, unpublished code was used in this work, it must be provided with the revision and access to it must be disclosed in the Code Availability Statement in the manuscript. Please also provide the completed software submission checklist. This form is a dynamic 'smart pdf' and must be downloaded and completed in Adobe Reader, instead of opening it in a web browser: https://www.nature.com/documents/nr-software-policy.pdf

If molecular dynamics (MD) simulations were performed, please refer to this Editorial and provide a completed MD simulations checklist with your revision: https://www.nature.com/articles/s42003-023-04653-0

Lastly, if it applies, please complete and upload the completed machine learning checklist with your submission for re-review: https://www.nature.com/documents/machine-learning-checklist.pdf

5. SOURCE GEL IMAGES: Unprocessed scans should be provided for all gels and western blots presented in figures and should be clearly labelled with the figure number in the file title. The source gel images should be uncropped, unmodified images, with molecular weight markers. Uncropped, unmodified gel images from existing experimental repeats, in addition to the data shown in the figures, can also be presented in source data. Please provide these files as PDF with your revision.

Please ensure that all control panels for gels and western blots are appropriately described as loading or IP controls. Lastly, please ensure that all images in the paper are checked for duplication of panels and for splicing of gel lanes.

6. SOURCE NUMERICAL DATA: we urge authors to provide, in tabular form in excel files, the data underlying the graphical representations used in figures. This is to further increase transparency in data reporting, as detailed in this editorial (http://www.nature.com/nsmb/journal/v22/n10/full/nsmb.3110.html). Please provide one excel file per figure max, with one panel per tab. When submitting files, the title field should indicate which figure the source data pertains to.

7. DATA AVAILABILITY: this journal strongly supports public availability of data. All data used in accepted papers should be available via a public data repository, or alternatively, as Supplementary Information. If data can only be shared on request, please explain why in your Data Availability Statement and in the correspondence with your editor.

For some data types, deposition in a public repository is mandatory as detailed below:

https://www.nature.com/nature-research/editorial-policies/reporting-standards#availability-of-data

Finally, please ensure that you retain unprocessed data and metadata files after publication, ideally archiving data in perpetuity, as these may be requested during the peer review and production process or after publication if any issues arise.

EXTENDED DATA FIGURES

When re-submitting your manuscript, please ensure that any supplementary figures and tables that are crucial to the manuscript's conclusions are converted into Extended Data figures and tables to increase visibility of these data. Extended Data figures and tables are online-only (present in the online PDF and full-text HTML versions of the paper), peer-reviewed display items that provide essential background to the article but are not included in the main article due to space constraints. A maximum of ten Extended Data display items (figures and tables) is permitted.

While we encourage the use of color in preparing figures, please note that this will incur a charge to partially defray the cost

of printing. Information about color charges can be found at http://www.nature.com/nsmb/authors/submit/index.html#costs

Nature Structural & Molecular Biology is committed to improving transparency in authorship. As part of our efforts in this direction, we are now requesting that all authors identified as 'corresponding author' on published papers create and link their Open Researcher and Contributor Identifier (ORCID) with their account on the Manuscript Tracking System (MTS), prior to acceptance. This applies to primary research papers only. ORCID helps the scientific community achieve unambiguous attribution of all scholarly contributions. You can create and link your ORCID from the home page of the MTS by clicking on 'Modify my Springer Nature account'. For more information please visit please visit www.springernature.com/orcid.

Please use the link below to submit your revised manuscript and related files:

Link Redacted

Note: This URL links to your confidential home page and associated information about manuscripts you may have submitted, or that you are reviewing for us. If you wish to forward this email to co-authors, please delete the link to your homepage.

We look forward to seeing the revised manuscript and thank you for the opportunity to review your work.

Sincerely,
Sara

Sara Osman, Ph.D.
Senior Editor
Nature Structural & Molecular Biology

Reviewers' Comments:

Reviewer #1 (Remarks to the Author):

Summary:

This work provides a rigorous benchmark of AI-based protein-ligand co-folding methods. The work is very timely and significant because AI-based protein-ligand co-folding methods have emerged as the most promising methods for protein-ligand structure prediction, which is important for drug design and discovery. The work made the following major contributions:

(1) It provides a very large dataset comprised of 2,600 high-resolution protein-ligand systems released after the training data cutoff date used by these methods to benchmark them. The results based on the large dataset are more statistically sound than on the small datasets in the field.
(2) It benchmarked four state-of-the-art methods recently developed in the field, including AlphaFold3, Chai-1, Protenix, and Boltz-1. The results can help users to choose how to use them.
(3) It introduces an interesting metric based on the product of binding pocket coverage and Combined Overlap Score (SuCOS) of the ligand pose to measure the similarity between test data and training data. This metric seemly works better than traditional metrics such as protein sequence identity or ligand similarity.
(4) The evaluation results convincingly show that the current AI-based co-folding methods still do not perform well on new protein pocket-ligand poses that are not similar to the training data. This finding can help the developers to design new methods to address the problem. This also help users understand how to use the predictions correctly.
(5) The evaluation results also demonstrate ranking predicted poses is still a major challenge that needs to be addressed.

The contributions above make the data, evaluation method, and results in this work highly valuable and significant to the field.

Below are two minor comments for the authors to consider to further improve the manuscript:

Minor comments:

(1) The benchmark dataset used in this work contains both single-ligand and multi-ligand systems. The multi-ligand subset is particularly interesting because there are few multi-ligand datasets in the field. Can you comment on if the co-folding methods work differently on single-ligand systems and multi-ligand systems? Some analysis or comparison of the performance on the two kinds of systems would be helpful.
(2) The major finding in this work is somewhat similar to what was found in another recent, independent study (https://arxiv.org/abs/2405.14108), i.e., the current protein-ligand pose prediction methods including co-folding ones work well on proteins/ligands similar to the training data but perform poorly on the ones dissimilar to the training data, even though

the experimental settings of the two studies (e.g., the definition of similarity and datasets) are very different. It may be helpful to discuss the similarity and difference between the two studies in this work to provide readers a complete picture of the study on this topic.

Reviewer #1 (Remarks on code availability):

The code is well organized.

Reviewer #2 (Remarks to the Author):

First of all, I would like to thank you for giving me the opportunity to review the manuscript "Have protein-ligand cofolding methods moved beyond memorisation?" by P. Skrinjar et al.

This study aims to rigorously evaluate the ability of modern deep learning "cofolding" methods to predict protein–ligand interactions—an essential step for advancing computational drug discovery. While deep learning has transformed protein structure prediction, extending these successes to protein–ligand complexes has remained challenging due to insufficient benchmarking datasets and protocols. To address this, the authors introduce Runs N' Poses, a new dataset of 2,600 high-resolution protein–ligand systems released after the training cutoff of existing models, and introduces relevent metrics to estimate the success rate of cofolding approaches in this context to measure or avoid data leakage. Using this benchmark, they systematically assess four state-of-the-art cofolding methods, revealing that current models often memorize ligand poses from training data rather than generalizing to novel systems.
The analysis shows that while protein modeling is accurate, ligand pose prediction remains unreliable, with performance tightly linked to training-set similarity. Errors arise from both inadequate sampling of correct poses and poor ranking of predicted conformations. The findings highlight data scarcity and bias as central obstacles, emphasizing the need for more diverse training data, improved evaluation metrics, and integration of physics-based and chemical priors. By providing an open and realistic benchmark, the study offers a foundation for fairer assessment and future development of more generalizable deep learning models for protein–ligand interaction prediction.

This manuscript is both timely and highly relevant, offering a crucial contribution to the field. In light of the current enthusiasm surrounding deep learning and the widespread media attention given to AlphaFold3—well deserved for its remarkable achievements in protein structure prediction—many researchers outside the computer-aided drug design (CADD) community have gained the mistaken impression that AlphaFold3 and similar tools have effectively solved the protein–ligand docking problem. This is clearly not the case. In fact, this overly optimistic perception has been further reinforced by misconfigured benchmarking practices that overlook data leakage between training and evaluation sets. Such benchmarks often fail to distinguish between genuine predictive power and the mere memorization of previously seen complexes. As a result, current cofolding–based approaches can typically approximate ligand poses only when highly similar protein–ligand systems were already present in the training data, rather than demonstrating true generalization to novel targets. The present study provides a much-needed, evidence-based perspective that corrects these misconceptions, grounding the discussion in rigorous scientific analysis and objective data rather than overly enthusiastic assumptions.

Overall, the manuscript is clearly written and structured. The introduction effectively situates the work within its broader scientific context, highlighting both its relevance and originality. The methods are described with sufficient detail to ensure reproducibility, and the availability of the underlying data represents a valuable resource that will enable further analysis and benchmarking by other research teams. The results are sound and statistically supported. Illustrations are clear and support the results. References are comprehensive.

Major concerns:
- The authors briefly mention (lines 185–195) that approximately 25% of the geometries predicted as correct by current cofolding approaches are, in fact, physically or chemically inconsistent, even in cases where the target system is highly similar to the training data. For this reviewer, this represents a critical limitation of cofolding-based methods. Supporting a drug design project with a docking or pose prediction tool requires accurate and physically meaningful molecular interactions between the ligand and the protein target—information that drives ligand optimization—rather than a superficial estimate of the ligand's location within the binding site. Consequently, the true success rate of these approaches for practical drug design applications is closer to 60–74%, rather than the 81–89% reported when such incorrect geometries are not filtered out (and when the studied complexes are very close to the training data). Although this issue was also addressed in the PoseBuster paper, it deserves stronger emphasis in the discussion, as it directly reinforces the authors' argument regarding the need to integrate more robust physics-based principles into deep learning cofolding models.
- In lines 450–460, the authors note that in some cases, the cofolding approaches produce incorrect configurations for asymmetric carbons, even when the correct chirality is explicitly defined in the input structure. This issue is particularly concerning for the use of such predictions in drug design, where stereochemistry plays a critical role in determining molecular recognition and binding affinity. Have the authors conducted a systematic analysis of ligand geometries to quantify the percentage of cases where stereochemical configurations were misassigned? Furthermore, have the authors examined if other types of chemical misrepresentations occur in the generated ligand structures—for instance, loss or misassignment of aromaticity, incorrect enforcement of aromaticity in aliphatic rings, or misinterpretation of double and triple bonds, etc? A more comprehensive assessment of these structural inaccuracies would provide valuable insight into the current limitations of cofolding models for reliable small-molecule modeling, and contribute to find rooms for improvements.

- In Figures 1A and 1B, the authors present a “Best” curve, obtained by aggregating the results from all cofolding approaches and selecting, for each case, the prediction closest to the experimental structure. This curve outperforms individual methods by approximately 5–20%, and the authors (lines 208–210) interpret this as evidence of a certain degree of complementarity among the models. While this interpretation is intriguing, it may be somewhat misleading. The concept of consensus docking indeed relies on combining complementary algorithms whose differing strengths can offset one another’s weaknesses. However, in the present study, the evaluated methods appear to be algorithmically and training-wise very similar, which limits the validity of invoking complementarity. Moreover, for systems with low similarity to the training data—where the success rate drops to 10–20%—such improvements could simply reflect statistical accumulation rather than true complementarity. Indeed, given the inherent constraints in binding site size and geometry, a 10–20% success rate can realistically be achieved even by a purely random docking algorithm that simply places ligands arbitrarily within the pocket. To illustrate, if one were to combine the outputs of ten very poor docking programs, each with a 10% success rate, the aggregated “best” outcome could misleadingly approach 100%. This would not reflect true complementarity between methods, but merely the statistical accumulation of random successes. In other words, even a set of random predictions could yield an apparently higher “best” success rate when enough attempts are aggregated. To use an analogy: following million lottery players increases the likelihood that one will win—not because their strategies are complementary, but because of the law of large numbers. Therefore, I recommend the authors adopt a more cautious interpretation of the “Best” curve, clearly distinguishing between complementarity and result accumulation effects.
• In lines 230–260, the authors note that, despite the addition of approximately 25,000 extra data points for training, the generalizability of Boltz-2 compared to Boltz-1 did not improve. They also suggest that increasing the amount of training data could help alleviate the issue of data scarcity, while acknowledging—rightly so, I think—that current architectures may not fully benefit from larger datasets due to algorithmic limitations. The discussion mentions several potential strategies, such as new data-generation initiatives or leveraging proprietary datasets from pharmaceutical companies. However, given the astronomical size of chemical space and the extreme structural diversity of drug-like molecules, the fact that an additional 25k data points failed to yield any measurable improvement raises a crucial question: would these initiatives truly be sufficient to achieve meaningful generalization? In line with this, is there a way to estimate or quantify how many diverse and representative data points would be required for current deep learning approaches to reach reliable predictive performance? Addressing this question, even approximately, would greatly help contextualize the feasibility of scaling data-driven solutions to overcome current limitations. Could the authors comment on this?

Minor concerns:
- Boltz-1 is mentioned in the abstract (line 087), but not Boltz-2 (while nother AF2 and AF3 are). This could be corrected.
- In Fig 1E., the % values written in white within the histogram bars are very difficult to read when printed.
- In Fig 2A., the legend in the Fig indicates that Boltz-1 is in red and Boltz-2 in green, while the figure caption mentioned the reverse. The legend or the caption should be corrected.

Reviewer #3 (Remarks to the Author):

This manuscript presents a valuable evaluation of four leading all-atom protein-ligand cofolding methods using the newly constructed Runs N’ Poses benchmark dataset (2,600 high-resolution systems post the 2021-09-30 training cutoff), effectively revealing the strong correlation between prediction accuracy and training set similarity—highlighting the "memorization" limitation of current cofolding approaches. The dataset design (controlling for training-test leakage via time cutoff) and evaluation metrics (SuCOS-pocket for similarity, combined RMSD < 2 Å and LDDT-PLI > 0.8 for success) are well-considered, providing meaningful insights for the field. However, several revisions are needed to enhance the work’s rigor and practical relevance:

1. The study only evaluates cofolding methods and lacks comparisons with physics-based molecular docking tools (e.g., Glide, GOLD) that rely on force fields/empirical scoring functions and inherently avoid training set memorization. Adding such controls would address two critical questions: (i) Whether the poor performance of cofolding methods on low SuCOS-pocket systems stems from true memorization or inherent high difficulty of these systems (if docking methods perform stably on low SuCOS-pocket systems, it confirms memorization; if docking also struggles, it indicates system difficulty). (ii) Whether traditional tools should be preferred for low SuCOS-pocket systems (a key scenario for de novo drug design), which would strengthen the manuscript’s practical guidance for drug discovery.

2. While SuCOS-pocket is effectively used to quantify training set similarity and reveal memorization, the manuscript does not clarify whether it can serve as an indicator for predicting the credibility of cofolding-derived complexes. For instance, if a predicted complex has low SuCOS-pocket similarity to known PDB entries, can this directly imply low credibility? Additional analysis is needed to address this: SuCOS-pocket should be contextualized with core accuracy metrics (RMSD, LDDT-PLI) and physical validity checks (PoseBusters, e.g., avoiding steric clashes as noted in the manuscript). For example, a low SuCOS-pocket score paired with RMSD < 2 Å and LDDT-PLI > 0.8 might indicate rare generalization success, rather than low credibility. Such discussion would prevent misinterpretation of SuCOS-pocket and enrich the metric’s utility.

3. Minorly, the Discussion section could better delineate the applicable scope of cofolding methods based on SuCOS-pocket ranges. For example, explicitly stating that cofolding methods are reliable for high SuCOS-pocket systems (where memorization is less problematic) but require caution for low SuCOS-pocket cases (unless paired with rigorous accuracy/physical checks) would help readers better navigate method selection.

Overall, these revisions would resolve ambiguities in the current conclusions, strengthen the work's comparative framework, and enhance its value for both method development and practical drug design. The core findings are significant, and the proposed changes are feasible given the existing Runs N' Poses dataset.

Reviewer #3 (Remarks on code availability):

The code and resources provided in the manuscript are well-positioned to support result reproducibility and community reuse, with comprehensive data access and transparent methodological documentation.

Version 1:

Decision Letter:

Our ref: NSMB-A51521A

21st Jan 2026

Dear Dr. Durairaj,

Thank you for submitting your revised manuscript "Have protein-ligand cofolding methods moved beyond memorisation?" (NSMB-A51521A). It has now been seen by the original referees and their comments are below. The reviewers find that the paper has improved in revision, and therefore we'll be happy in principle to publish it in Nature Structural & Molecular Biology, pending minor revisions to satisfy the referees' final requests and to comply with our editorial and formatting guidelines.

We are now performing detailed checks on your paper and will send you a checklist detailing our editorial and formatting requirements in the next couple of weeks. Please do not upload the final materials and make any revisions until you receive this additional information from us.

To facilitate our work at this stage, it is important that we have a copy of the main text as a word file. If you could please send along a word version of this file as soon as possible, we would greatly appreciate it; please make sure to copy the NSMB account (cc'ed above).

Thank you again for your interest in Nature Structural & Molecular Biology Please do not hesitate to contact me if you have any questions.

Sincerely,
Sara

Sara Osman, Ph.D.
Senior Editor
Nature Structural & Molecular Biology

Reviewer #1 (Remarks to the Author):

The revised manuscript addresses all of my review comments very well and represents a highly valuable contribution to the protein–ligand modeling field.

Reviewer #1 (Remarks on code availability):

The code is well organized and well documented.

Reviewer #2 (Remarks to the Author):

Dear Editor,

The authors have satisfactorily addressed all my questions. I have no further questions to ask or points to raise.

Sincerely,

Reviewer #3 (Remarks to the Author):

I find that the authors have substantially addressed my first concern by adding a comprehensive comparison with a physics-based docking method (GLIDE) and by clarifying how these baselines behave across different SuCOS-pocket similarity regimes. To further strengthen this part, I recommend that the authors also cite the recently published study in Proc.

Natl. Acad. Sci. U.S.A. 122 (52) e2511925122, which explicitly demonstrates how incorporating binding-site side-chain flexibility can improve docking performance on challenging targets. Including this reference in the new Section 2.6 and/or the Discussion would better situate the GLIDE analysis within the broader literature on flexible docking and provide readers with a concrete example of how pocket flexibility can be exploited in practice.
Apart from this minor addition, I am satisfied with the authors’ responses and the corresponding revisions. I therefore recommend acceptance after minor revision.

Reviewer #3 (Remarks on code availability):

The code and resources provided in the manuscript are well-positioned to support result reproducibility and community reuse, with comprehensive data access and transparent methodological documentation.

Version 2:

Decision Letter:

25th Mar 2026

Dear Dr. Durairaj,

We are now happy to accept your revised paper "Evaluating generalisation in protein-ligand cofolding methods" for publication as an Article in Nature Structural & Molecular Biology.

Acceptance is conditional on the manuscript's not being published elsewhere and on there being no announcement of this work to the newspapers, magazines, radio or television until the publication date in Nature Structural & Molecular Biology.

Over the next few weeks, your paper will be copyedited to ensure that it conforms to Nature Structural & Molecular Biology style. Once your paper is typeset, you will receive an email with a link to choose the appropriate publishing options for your paper and our Author Services team will be in touch regarding any additional information that may be required.

After the grant of rights is completed, you will receive a link to your electronic proof via email with a request to make any corrections within 48 hours. If, when you receive your proof, you cannot meet this deadline, please inform us at rjsproduction@springernature.com immediately.

You will not receive your proofs until the publishing agreement has been received through our system.

Due to the importance of these deadlines, we ask that you please let us know now whether you will be difficult to contact over the next month. If this is the case, we ask you provide us with the contact information (email, phone and fax) of someone who will be able to check the proofs on your behalf, and who will be available to address any last-minute problems.

To assist our authors in disseminating their research to the broader community, our SharedIt initiative provides all co-authors with the ability to generate a unique shareable link that will allow anyone (with or without a subscription) to read the published article. Recipients of the link with a subscription will also be able to download and print the PDF.

As soon as your article is published, you can generate your shareable link by entering the DOI of your article here: http://authors.springernature.com/share. Corresponding authors will also receive an automated email with the shareable link

Note the policy of the journal on data deposition: http://www.nature.com/authors/policies/availability.html.

Your paper will be published online soon after we receive proof corrections and will appear in print in the next available issue. You can find out your date of online publication by contacting the production team shortly after sending your proof corrections.

You may wish to make your media relations office aware of your accepted publication, in case they consider it appropriate to organize some internal or external publicity. Once your paper has been scheduled you will receive an email confirming the publication details. This is normally 3-4 working days in advance of publication. If you need additional notice of the date and time of publication, please let the production team know when you receive the proof of your article to ensure there is sufficient time to coordinate. Further information on our embargo policies can be found here: https://www.nature.com/authors/policies/embargo.html

You can now use a single sign-on for all your accounts, view the status of all your manuscript submissions and reviews, access usage statistics for your published articles and download a record of your refereeing activity for the Nature journals.

If you have not already done so, we strongly recommend that you upload the step-by-step protocols used in this manuscript

to the Protocol Exchange. Protocol Exchange is an open online resource that allows researchers to share their detailed experimental know-how. All uploaded protocols are made freely available, assigned DOIs for ease of citation and fully searchable through nature.com. Protocols can be linked to any publications in which they are used and will be linked to from your article. You can also establish a dedicated page to collect all your lab Protocols. By uploading your Protocols to Protocol Exchange, you are enabling researchers to more readily reproduce or adapt the methodology you use, as well as increasing the visibility of your protocols and papers. Upload your Protocols at www.nature.com/protocolexchange/. Further information can be found at www.nature.com/protocolexchange/about.

An online order form for reprints of your paper is available at https://www.nature.com/reprints/author-reprints.html. Please let your coauthors and your institutions' public affairs office know that they are also welcome to order reprints by this method.

Authors may need to take specific actions to achieve compliance with funder and institutional open access mandates. If your research is supported by a funder that requires immediate open access (e.g. according to Plan S principles or the NIH public access policy) then you should select the gold OA route, and we will direct you to the compliant route where possible. Because authors warrant under our subscription licensing terms that they haven't committed to licensing any version of their article under a licence inconsistent with the terms of our agreement – including the applicable embargo period – publication under the subscription model isn't suitable for authors whose funders require no embargo.

In approximately 10 business days you will receive an email with a link to choose the appropriate publishing options for your paper and our Author Services team will be in touch regarding any additional information that may be required.

You will not receive your proofs until the publishing agreement has been received through our system.

If you have any questions about our publishing options, costs, Open Access requirements, or our legal forms, please contact ASJournals@springernature.com

Sincerely,
Sara

Sara Osman, Ph.D.
Senior Editor
Nature Structural & Molecular Biology

Click here if you would like to recommend Nature Structural & Molecular Biology to your librarian:
http://www.nature.com/subscriptions/recommend.html#forms

Open Access This Peer Review File is licensed under a Creative Commons Attribution 4.0 International License, which

permits use, sharing, adaptation, distribution and reproduction in any medium or format, as long as you give appropriate credit to the original author(s) and the source, provide a link to the Creative Commons license, and indicate if changes were made.
In cases where reviewers are anonymous, credit should be given to 'Anonymous Referee' and the source.
The images or other third party material in this Peer Review File are included in the article's Creative Commons license, unless indicated otherwise in a credit line to the material. If material is not included in the article's Creative Commons license and your intended use is not permitted by statutory regulation or exceeds the permitted use, you will need to obtain permission directly from the copyright holder.
To view a copy of this license, visit https://creativecommons.org/licenses/by/4.0/

We thank you for the time and effort dedicated to reviewing our manuscript. We have carefully considered the feedback provided by all three reviewers. In response to these comments, we have strengthened the discussion and performed additional analyses, including a more granular analysis of PoseBusters checks and a comprehensive comparison with physics-based docking (GLIDE). We believe these revisions have greatly strengthened the quality and rigor of the manuscript. Please find the detailed responses provided point-by-point below.

Reviewers' Comments:

Reviewer #1 (Remarks to the Author):

Summary:

This work provides a rigorous benchmark of AI-based protein-ligand co-folding methods. The work is very timely and significant because AI-based protein-ligand co-folding methods have emerged as the most promising methods for protein-ligand structure prediction, which is important for drug design and discovery. The work made the following major contributions:

(1) It provides a very large dataset comprised of 2,600 high-resolution protein-ligand systems released after the training data cutoff date used by these methods to benchmark them. The results based on the large dataset are more statistically sound than on the small datasets in the field.
(2) It benchmarked four state-of-the-art methods recently developed in the field, including AlphaFold3, Chai-1, Protenix, and Boltz-1. The results can help users to choose how to use them.
(3) It introduces an interesting metric based on the product of binding pocket coverage and Combined Overlap Score (SuCOS) of the ligand pose to measure the similarity between test data and training data. This metric seemly works better than traditional metrics such as protein sequence identity or ligand similarity.
(4) The evaluation results convincingly show that the current AI-based co-folding methods still do not perform well on new protein pocket-ligand poses that are not similar to the training data. This finding can help the developers to design new methods to address the problem. This also help users understand how to use the predictions correctly.
(5) The evaluation results also demonstrate ranking predicted poses is still a major challenge that needs to be addressed.

The contributions above make the data, evaluation method, and results in this work highly valuable and significant to the field.

Below are two minor comments for the authors to consider to further improve the manuscript:

Minor comments:

(1) The benchmark dataset used in this work contains both single-ligand and multi-ligand systems. The multi-ligand subset is particularly interesting because there are few multi-ligand datasets in the field. Can you comment on if the co-folding methods work differently on single-ligand systems and multi-ligand systems? Some analysis or comparison of the performance on the two kinds of systems would be helpful.

Answer:
We thank the reviewer for their positive assessment. In the manuscript, we also evaluated the performance of cofolding methods separately on single-ligand systems (**Supplementary Fig. S3I**) and multi-ligand systems (**Supplementary Fig. S3J**). For both sub-tasks, we observed the same overall pattern of memorisation, as noted in **lines 338–341**. While performance in the lowest similarity bin appears relatively higher for multi-ligand systems compared to single-ligand systems, the limited number of data points in this bin and the inclusion of cofactor analogs (discussed in **Section 2.3**) for the multi-ligand set prevents us from drawing this conclusion.

(2) The major finding in this work is somewhat similar to what was found in another recent, independent study (https://arxiv.org/abs/2405.14108), i.e., the current protein-ligand pose prediction methods including co-folding ones work well on proteins/ligands similar to the training data but perform poorly on the ones dissimilar to the training data, even though the experimental settings of the two studies (e.g., the definition of similarity and datasets) are very different. It may be helpful to discuss the similarity and difference between the two studies in this work to provide readers a complete picture of the study on this topic.

Answer:
We appreciate the reviewer's comment regarding the parallels with the recent PoseBench study. We would like to clarify that the convergence of findings is largely expected, as the PoseBench authors adopted the Runs N' Poses SuCOS-pocket similarity framework (from our preprint released in Feb 2025) to categorise their dataset. Furthermore, as PoseBench uses a subset of PoseBusters, which follows dataset procurement criteria similar to ours, the dataset composition is comparable. We have added a statement to the manuscript (**lines 828-831**) to showcase the broader applicability of the similarity metric developed in this work and cite studies where it and the dataset have already been used.

Reviewer #1 (Remarks on code availability):

The code is well organized.

Reviewer #2 (Remarks to the Author):

First of all, I would like to thank you for giving me the opportunity to review the manuscript "Have protein-ligand cofolding methods moved beyond memorisation?" by P. Skrinjar et al.

This study aims to rigorously evaluate the ability of modern deep learning "cofolding" methods to predict protein–ligand interactions—an essential step for advancing computational drug discovery. While deep learning has transformed protein structure prediction, extending these successes to protein–ligand complexes has remained challenging due to insufficient benchmarking datasets and protocols. To address this, the authors introduce Runs N' Poses, a new dataset of 2,600 high-resolution protein–ligand systems released after the training cutoff of existing models, and introduces relevent metrics to estimate the success rate of cofolding approaches in this context to measure or avoid data leakage. Using this benchmark, they systematically assess four state-of-the-art cofolding methods, revealing that current models often memorize ligand poses from training data rather than generalizing to novel systems.
The analysis shows that while protein modeling is accurate, ligand pose prediction remains unreliable, with performance tightly linked to training-set similarity. Errors arise from both inadequate sampling of correct poses and poor ranking of predicted conformations. The findings highlight data scarcity and bias as central obstacles, emphasizing the need for more diverse training data, improved evaluation metrics, and integration of physics-based and chemical priors. By providing an open and realistic benchmark, the study offers a foundation for fairer assessment and future development of more generalizable deep learning models for protein–ligand interaction prediction.

This manuscript is both timely and highly relevant, offering a crucial contribution to the field. In light of the current enthusiasm surrounding deep learning and the widespread media attention given to AlphaFold3—well deserved for its remarkable achievements in protein structure prediction—many researchers outside the computer-aided drug design (CADD) community have gained the mistaken impression that AlphaFold3 and similar tools have effectively solved the protein–ligand docking problem. This is clearly not the case. In fact, this overly optimistic perception has been further reinforced by misconfigured benchmarking practices that overlook data leakage between training and evaluation sets. Such benchmarks often fail to distinguish between genuine predictive power and the mere memorization of previously seen complexes.

As a result, current cofolding–based approaches can typically approximate ligand poses only when highly similar protein–ligand systems were already present in the training data, rather than demonstrating true generalization to novel targets. The present study provides a much-needed, evidence-based perspective that corrects these misconceptions, grounding the discussion in rigorous scientific analysis and objective data rather than overly enthusiastic assumptions.

Overall, the manuscript is clearly written and structured. The introduction effectively situates the work within its broader scientific context, highlighting both its relevance and originality. The methods are described with sufficient detail to ensure reproducibility, and the availability of the underlying data represents a valuable resource that will enable further analysis and benchmarking by other research teams. The results are sound and statistically supported. Illustrations are clear and support the results. References are comprehensive.

Major concerns:

- The authors briefly mention (lines 185–195) that approximately 25% of the geometries predicted as correct by current cofolding approaches are, in fact, physically or chemically inconsistent, even in cases where the target system is highly similar to the training data. For this reviewer, this represents a critical limitation of cofolding-based methods. Supporting a drug design project with a docking or pose prediction tool requires accurate and physically meaningful molecular interactions between the ligand and the protein target—information that drives ligand optimization—rather than a superficial estimate of the ligand's location within the binding site. Consequently, the true success rate of these approaches for practical drug design applications is closer to 60–74%, rather than the 81–89% reported when such incorrect geometries are not filtered out (and when the studied complexes are very close to the training data). Although this issue was also addressed in the PoseBuster paper, it deserves stronger emphasis in the discussion, as it directly reinforces the authors' argument regarding the need to integrate more robust physics-based principles into deep learning cofolding models.

Answer:

We thank the reviewer for their positive assessment and this insightful comment. We have now included the following lines in the Discussion (**lines 843-849**) to emphasize the point about the methods not being useful in drug discovery campaigns unless physical implausibilities are addressed:

"The results also show that the physical plausibility of generated poses are sometimes suboptimal, again precluding usage in real-world drug discovery campaigns. Promisingly, the

diffusion steering strategy implemented in Boltz-1x largely solves these issues, and could straightforwardly be implemented into other cofolding methods using a similar architecture. However, this still does not change the generalisation trend, still calling for concerted improvements in architecture and training to address memorisation."

- In lines 450–460, the authors note that in some cases, the cofolding approaches produce incorrect configurations for asymmetric carbons, even when the correct chirality is explicitly defined in the input structure. This issue is particularly concerning for the use of such predictions in drug design, where stereochemistry plays a critical role in determining molecular recognition and binding affinity. Have the authors conducted a systematic analysis of ligand geometries to quantify the percentage of cases where stereochemical configurations were misassigned? Furthermore, have the authors examined if other types of chemical misrepresentations occur in the generated ligand structures—for instance, loss or misassignment of aromaticity, incorrect enforcement of aromaticity in aliphatic rings, or misinterpretation of double and triple bonds, etc? A more comprehensive assessment of these structural inaccuracies would provide valuable insight into the current limitations of cofolding models for reliable small-molecule modeling, and contribute to find rooms for improvements.

Answer:
We thank the reviewer for pointing out that a more systematic analysis of chemical misrepresentations should be done. We addressed this point in **Supplementary Figure 1E**, where we performed an in-depth analysis of chemical misrepresentations using the "redock" mode of the PoseBusters suite which include the misrepresentations mentioned by the reviewer. The figure shows the percentage of systems passing each PoseBusters check across all predicted systems for each method. We present our results and conclusions for this part in **lines 216-236**, where we find that including the physics-inspired potentials for diffusion steering at inference time, as is done by Boltz-1x, helped with resolving the majority of these chemical implausibilities.

- In Figures 1A and 1B, the authors present a "Best" curve, obtained by aggregating the results from all cofolding approaches and selecting, for each case, the prediction closest to the experimental structure. This curve outperforms individual methods by approximately 5–20%, and the authors (lines 208–210) interpret this as evidence of a certain degree of complementarity among the models. While this interpretation is intriguing, it may be somewhat misleading. The concept of consensus docking indeed relies on combining complementary algorithms whose differing strengths can offset one another's weaknesses. However, in the present study, the evaluated methods appear to be algorithmically and training-wise very similar, which limits the validity of invoking complementarity. Moreover, for systems with low

similarity to the training data—where the success rate drops to 10–20%—such improvements could simply reflect statistical accumulation rather than true complementarity. Indeed, given the inherent constraints in binding site size and geometry, a 10–20% success rate can realistically be achieved even by a purely random docking algorithm that simply places ligands arbitrarily within the pocket. To illustrate, if one were to combine the outputs of ten very poor docking programs, each with a 10% success rate, the aggregated "best" outcome could misleadingly approach 100%. This would not reflect true complementarity between methods, but merely the statistical accumulation of random successes. In other words, even a set of random predictions could yield an apparently higher "best" success rate when enough attempts are aggregated. To use an analogy: following million lottery players increases the likelihood that one will win—not because their strategies are complementary, but because of the law of large numbers. Therefore, I recommend the authors adopt a more cautious interpretation of the "Best" curve, clearly distinguishing between complementarity and result accumulation effects.

Answer:

We thank the reviewer for this comment and we agree that our interpretation of the "best" curve might be misleading. Consequently, we have removed the "Best" curve from all figures except Figure 1 to prevent misleading interpretations. We have retained the gray line in Figures 1A and 1B solely to illustrate that AlphaFold3 is not the top performer on every single system, but we have rephrased the accompanying text (**lines 204-206, quoted below**) to avoid implying that this represents complementarity. We note that the statistical accumulation effect is instead studied in more detail in Section 2.5.

"However, as indicated by the gray line representing the best-scored of the top-ranked models across all four methods (Figure 1A and 1B), AlphaFold3 is not the best-performing method on every system evaluated."

• In lines 230–260, the authors note that, despite the addition of approximately 25,000 extra data points for training, the generalizability of Boltz-2 compared to Boltz-1 did not improve. They also suggest that increasing the amount of training data could help alleviate the issue of data scarcity, while acknowledging—rightly so, I think—that current architectures may not fully benefit from larger datasets due to algorithmic limitations. The discussion mentions several potential strategies, such as new data-generation initiatives or leveraging proprietary datasets from pharmaceutical companies. However, given the astronomical size of chemical space and the extreme structural diversity of drug-like molecules, the fact that an additional 25k data points failed to yield any measurable improvement raises a crucial question: would these initiatives truly be sufficient to achieve meaningful generalization? In line with this, is there a way to estimate or quantify how many diverse and representative data points would be

required for current deep learning approaches to reach reliable predictive performance? Addressing this question, even approximately, would greatly help contextualize the feasibility of scaling data-driven solutions to overcome current limitations. Could the authors comment on this?

Answer:

We agree with the reviewer that current cofolding architectures are likely suboptimal for this task and we try to emphasize that in **lines 786-804**. Regarding the reviewer's question on quantifying the necessary data size: this is linked to a model's inductive bias, a model that effectively learns physical priors should theoretically require less data points. We believe it would be premature to conclude that additional data would not lead to improvements, especially given better architectures or modifications to current architectures, as reliably estimating the effect of further data scaling is extremely challenging and beyond the scope of this work. Data ablation experiments such as what OpenFold (DOI: 10.1038/s41592-024-02272-z) performed for the AlphaFold2 architecture could help answer such questions once a more robust architecture comes into play.
That being said, there is an on-going experiment with ApherisFold (https://www.apheris.com/resources/blog/apheris-launches-apherisfold-to-make-openfold3-securely-usable-in-pharma-environm), where the data from pharmaceutical companies is being used to train OpenFold3. This type of experiment could potentially answer some of these questions, especially if the data used covers unexplored parts of the chemical space. Identifying the optimal metric to guide the procurement of high-value training targets is also an ongoing challenge that is likely specific to the architecture being trained.

Minor concerns:
- Boltz-1 is mentioned in the abstract (line 087), but not Boltz-2 (while nother AF2 and AF3 are). This could be corrected.
Corrected, thank you.
- In Fig 1E., the % values written in white within the histogram bars are very difficult to read when printed.
Font size increased, thank you.
- In Fig 2A., the legend in the Fig indicates that Boltz-1 is in red and Boltz-2 in green, while the figure caption mentioned the reverse. The legend or the caption should be corrected.
Corrected, thank you.

Reviewer #3 (Remarks to the Author):

This manuscript presents a valuable evaluation of four leading all-atom protein-ligand cofolding methods using the newly constructed Runs N' Poses benchmark dataset (2,600 high-resolution systems post the 2021-09-30 training cutoff), effectively revealing the strong correlation between prediction accuracy and training set similarity—highlighting the "memorization" limitation of current cofolding approaches. The dataset design (controlling for training-test leakage via time cutoff) and evaluation metrics (SuCOS-pocket for similarity, combined RMSD < 2 Å and LDDT-PLI > 0.8 for success) are well-considered, providing meaningful insights for the field. However, several revisions are needed to enhance the work's rigor and practical relevance:

1. The study only evaluates cofolding methods and lacks comparisons with physics-based molecular docking tools (e.g., Glide, GOLD) that rely on force fields/empirical scoring functions and inherently avoid training set memorization. Adding such controls would address two critical questions: (i) Whether the poor performance of cofolding methods on low SuCOS-pocket systems stems from true memorization or inherent high difficulty of these systems (if docking methods perform stably on low SuCOS-pocket systems, it confirms memorization; if docking also struggles, it indicates system difficulty). (ii) Whether traditional tools should be preferred for low SuCOS-pocket systems (a key scenario for de novo drug design), which would strengthen the manuscript's practical guidance for drug discovery.

Answer:
We thank the reviewer for their positive assessment and this valuable suggestion. We agree that comparison with physics-based methods is an important and underexplored aspect in this context. To address this, we have added a comprehensive comparison with GLIDE in a new section (**Section 2.6**) and detailed the baseline construction in **Methods Section 5.3**.

Our findings address the reviewer's specific questions as follows:

(i) We found that systems in the lowest similarity bins are not inherently more difficult from a physics perspective. GLIDE redocking performed equally well on these low-similarity systems as it did on high-similarity ones. This reinforces our conclusion that the poor performance of cofolding methods in this regime is driven by a lack of training data, rather than the physical complexity of the binding site.

(ii) We evaluated docking performance under various "prior knowledge" scenarios. In realistic settings (where the exact holo-structure is unknown), traditional docking methods performed as poorly in the low-similarity bins as cofolding methods. This highlights that accurate protein pocket conformation prediction remains the primary bottleneck for both approaches.

We also note that these results represent automated baselines. They do not fully capture real-world drug discovery campaigns, where expert computational chemists combine template and pocket selection, receptor flexibility, and manual pose inspection to improve results. Developing more optimised automated pipelines is an exciting avenue for future work that we hope will benefit from the data and findings presented here. We have integrated the answers to the reviewer questions into the main text in **Section 2.6 and Discussion lines 821-824**.

2. While SuCOS-pocket is effectively used to quantify training set similarity and reveal memorization, the manuscript does not clarify whether it can serve as an indicator for predicting the credibility of cofolding-derived complexes. For instance, if a predicted complex has low SuCOS-pocket similarity to known PDB entries, can this directly imply low credibility? Additional analysis is needed to address this: SuCOS-pocket should be contextualized with core accuracy metrics (RMSD, LDDT-PLI) and physical validity checks (PoseBusters, e.g., avoiding steric clashes as noted in the manuscript). For example, a low SuCOS-pocket score paired with RMSD < 2 Å and LDDT-PLI > 0.8 might indicate rare generalization success, rather than low credibility. Such discussion would prevent misinterpretation of SuCOS-pocket and enrich the metric's utility.

Answer:

We thank the reviewer for this interesting suggestion. We explored the possibility of using SuCOS-pocket as a confidence indicator for predicted complexes, but we concluded that it is not a reliable metric for credibility due to both theoretical and technical limitations.

Theoretical Limitation: A high SuCOS-pocket similarity between a predicted complex and the training set does not necessarily have to correlate with accuracy, and indeed would not in a pure memorisation scenario. A model may predict a complex that strongly resembles a specific training system with a similar protein/ligand (resulting in a high similarity score) but with a completely incorrect pose (i.e high RMSD/low LDDT-PLI) for the target system of interest. In such cases, the metric would falsely indicate high credibility.

Technical Limitations: We faced challenges in implementing this analysis rigorously due to the output formats of current methods. Cofolding tools produce CIF files without bond connectivity, making reliable conversion to the valid SDF formats required for RDKit analysis extremely difficult and error-prone. Furthermore, we found that RDKit's shape/color and SuCOS functions are highly sensitive to chemical correctness, likely even stricter than PoseBusters, and frequently assign low scores to geometrically/visually near-identical poses.

To address the reviewer's comment, we performed a preliminary analysis using Boltz-1x (to minimize the molecule parsing issues) on a subset of systems. The resulting plot (shown below) compares the SuCOS-pocket similarity of the predicted complex (X-axis) against that of the ground truth (Y-axis). As expected, we observe a number of cases in the top-left quadrant where the ground truth is far from the training set, yet the model returns a prediction that strongly resembles a training system. Overall, the predictive power of this metric was low, performing slightly worse than ligand-chain pair iPTM. However, we cannot be 100% sure of these scores due to the incompatibility of file formats and outputs involved. Given these uncertainties and the effort involved to address them, we believe that developing a robust confidence score based on training similarity is a separate challenge that falls outside the scope of this work.

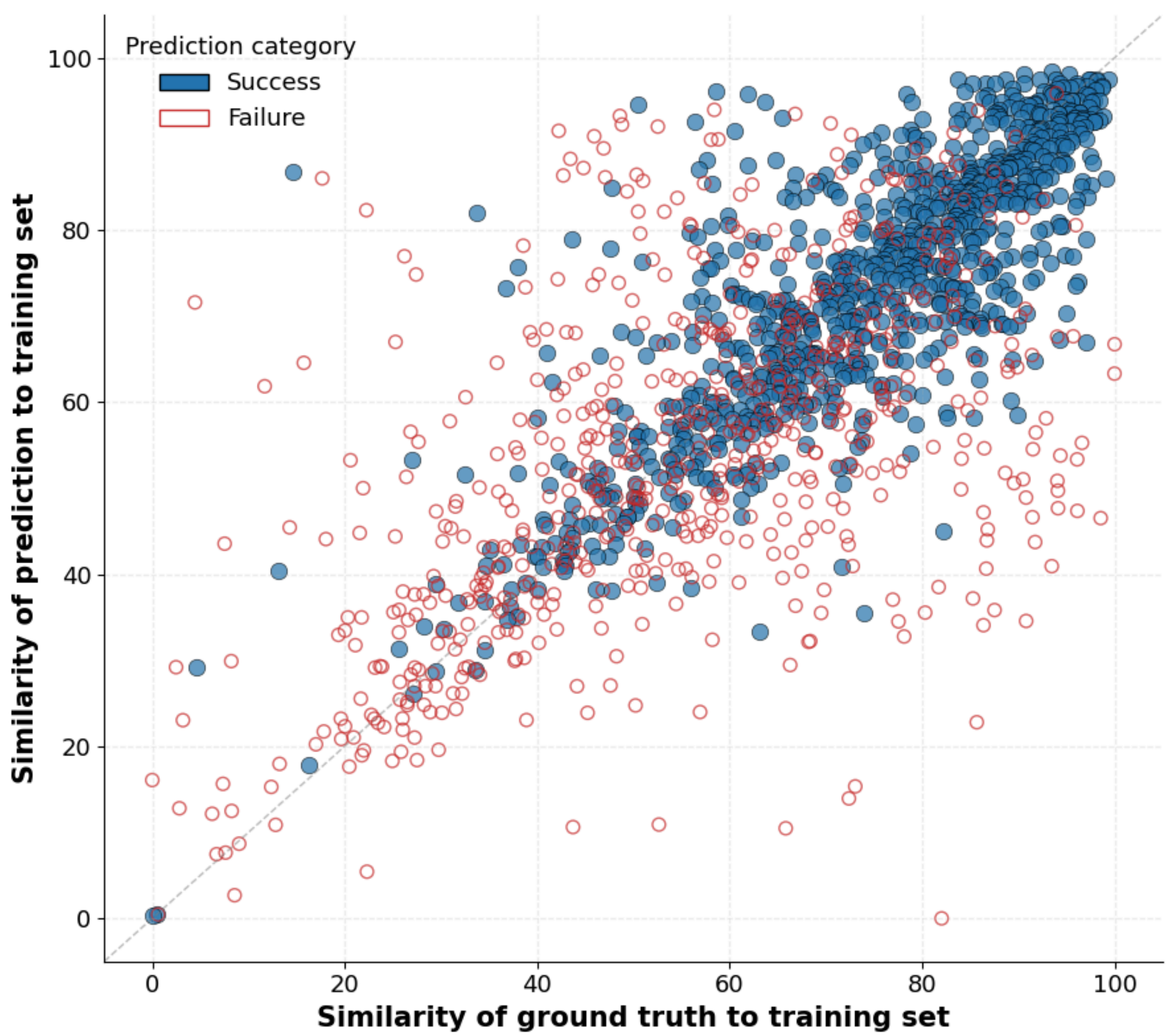

3. Minorly, the Discussion section could better delineate the applicable scope of cofolding methods based on SuCOS-pocket ranges. For example, explicitly stating that cofolding methods

are reliable for high SuCOS-pocket systems (where memorization is less problematic) but require caution for low SuCOS-pocket cases (unless paired with rigorous accuracy/physical checks) would help readers better navigate method selection.

Answer:

We thank the reviewer for this suggestion. We have expanded the Discussion (**lines 815–824**) to address this comment. We would like to note that while cofolding methods generally perform better on systems with high training-set similarity, they cannot be blindly trusted even in these scenarios due to the non-robustness to small changes in the ligand or binding pocket chemistry (as exemplified in **Figure 5**). This limitation was also observed in concurrent work by Masters et al. (2025), where mutations that should disrupt binding were overlooked.

The added text now reads:

"While it is easy to conclude that cofolding methods can in general be trusted on the cases where similar complexes have been seen in the PDB, our failure mode examples demonstrate that caution is still required even in this scenario, as small changes in ligand chemistry can lead to changes in poses which cofolding methods would likely not capture and instead would confidently return previously seen results. This was also seen in concurrent work where mutations in the binding pocket which should disrupt ligand binding were ignored. However, we find that applying physics-based docking methods for ligand placement also requires care as the results are heavily dependent on the accuracy of the protein and pocket conformation used."

Overall, these revisions would resolve ambiguities in the current conclusions, strengthen the work's comparative framework, and enhance its value for both method development and practical drug design. The core findings are significant, and the proposed changes are feasible given the existing Runs N' Poses dataset.

Reviewer #3 (Remarks on code availability):

The code and resources provided in the manuscript are well-positioned to support result reproducibility and community reuse, with comprehensive data access and transparent methodological documentation.

Reviewer #1:
Remarks to the Author:
The revised manuscript addresses all of my review comments very well and represents a highly valuable contribution to the protein–ligand modeling field.

Remarks on code availability:
The code is well organized and well documented.

Answer: We thank the reviewer for their positive assessment

Reviewer #4:
Remarks to the Author:
Dear Editor,

The authors have satisfactorily addressed all my questions. I have no further questions to ask or points to raise.

Sincerely,

Answer: We thank the reviewer for their positive assessment

Reviewer #5:
Remarks to the Author:
I find that the authors have substantially addressed my first concern by adding a comprehensive comparison with a physics-based docking method (GLIDE) and by clarifying how these baselines behave across different SuCOS-pocket similarity regimes. To further strengthen this part, I recommend that the authors also cite the recently published study in Proc. Natl. Acad. Sci. U.S.A. 122 (52) e2511925122, which explicitly demonstrates how incorporating binding-site side-chain flexibility can improve docking performance on challenging targets. Including this reference in the new Section 2.6 and/or the Discussion would better situate the GLIDE analysis within the broader literature on flexible docking and provide readers with a concrete example of how pocket flexibility can be exploited in practice.
Apart from this minor addition, I am satisfied with the authors' responses and the corresponding revisions. I therefore recommend acceptance after minor revision.

Remarks on code availability:
The code and resources provided in the manuscript are well-positioned to support result reproducibility and community reuse, with comprehensive data access and transparent methodological documentation.

Answer: We thank the reviewer for their positive assessment. We have addressed the comment by including the recommended reference in Section 2.6 and in the Discussion. In addition, we have incorporated an explanatory sentence highlighting how the incorporation of binding-site side-chain flexibility can improve docking performance.
